# New Pyrazolium Salts as a Support for Ionic Liquid Crystals and Ionic Conductors

**DOI:** 10.3390/ma11040548

**Published:** 2018-04-03

**Authors:** María Jesús Pastor, Ignacio Sánchez, José A. Campo, Rainer Schmidt, Mercedes Cano

**Affiliations:** 1 Dpto. Química Inorgánica, Fac. CC. Químicas, Universidad Complutense, E-28040 Madrid, Spain; mjpastor@ucm.es (M.J.P.); ismartinez@quim.ucm.es (I.S.); jacampo@ucm.es (J.A.C.); 2 Dpto. Física de Materiales, Fac. CC. Físicas, GFMC, Universidad Complutense, E-28040 Madrid, Spain; rainer.schmidt@fis.ucm.es

**Keywords:** pyrazolium salts, liquid crystals, ionic liquid crystals, smectic mesophase, ionic conductivity

## Abstract

Ionic liquid crystals (ILCs) are a class of materials that combine the properties of liquid crystals (LCs) and ionic liquids (ILs). This type of materials is directed towards properties such as conductivity in ordered systems at different temperatures. In this work, we synthesize five new families of ILCs containing symmetrical and unsymmetrical substituted pyrazolium cations, with different alkyl long-chains, and anions such as Cl^−^, BF_4_^−^, ReO_4_^−^, *p*-CH_3_-_6_H_4_SO_3_^−^ (PTS) and CF_3_SO_3_^−^ (OTf). We study their thermal behavior by polarized light optical microscopy (POM) and differential scanning calorimetry (DSC). All of them, except those with OTf as counteranion, show thermotropic mesomorphism. The observations by POM reveal textures of lamellar mesophases. Those agree with the arrangement observed in the X-ray crystal structure of [H_2_pz^R(4),R(4)^][ReO_4_]. The nature of the mesophases is also confirmed by variable temperature powder X-ray diffraction. On the other hand, the study of the dielectric properties at variable temperature in mesomorphic (Cl^−^ and BF_4_^−^) and non-mesomorphic (OTf) salts indicates that the supramolecular arrangement of the mesophase favors a greater ionic mobility and therefore ionic conductivity.

## 1. Introduction

Ionic liquid crystals (ILCs) have evoked considerable interest because they combine the properties of liquid crystals (LCs), such as dynamic molecular order, anisotropy, self-assembly and mesomorphism, with the properties of ionic liquids (ILs), such as ionic conductivity, low vapor pressure and high thermal stability [1,2].

These materials can exhibit interesting properties such as optical, magnetic and conductivity, among others [2,3,4,5], which make them highly interesting for potential application in synthesis and catalysis [6], as conductive materials [7,8,9], and as materials for energy and biology applications [10,11,12,13,14,15]. In particular, as conductive materials, they were proven to be useful in current information technology. It has also been proven that conductivity of ILCs can be modified and modulated by introducing those compounds into surface-treated porous membranes, being dependent on the molecular alignment on the pore walls [16]. In the same way, polymerizable ILCs have been used for fabrication of ion-conducting membranes [17].

In addition, silica-supported ILCs have also been used to build advanced materials for mesoporous structures of interest such as gas phase catalyst [18,19], and several materials have also been prepared showing interesting properties as high conductivity and compatibility with the organic media [1,2]. Several of those materials were used as a support of mesoporous structures.

However, from the best known application of LCs, that is, in liquid-crystal displays (LCDs), ILCs are very interesting materials for switching purposes as anisotropic nanostructured ion-conductive low-dimensional materials avoiding the inconveniences of LCs switching [2]. Then, one of the most important interests of the current research on ILCs is directed towards the achievement of ion-conductive materials, in which the ions can act as mobile charge carriers with one or two-dimensional ion-conduction, which should be related to the type of mesophase. In particular, columnar mesophases are of interest for 1D ion-conduction in the direction of columnar axes, while smectic LCs can be created for 2D ion-conductors [2].

Therefore, looking for well-ordered ionic groups, the ILCs materials in which the ionic moieties are assembled in regular ordered structures are expected to be efficient to work as ionic-conductors. Then, the research for stable ILCs should be necessary to produce advanced functional materials useful for interesting applications in modern electronic devices.

ILC materials previously investigated include a variety of salts containing cations such as triazolium, ammonium, pyrrolidinium, phosphonium [20,21,22,23], pyridinium [24,25,26] and especially imidazolium [9,27,28,29,30,31,32], where the cation acts as the mesogenic unit. It has been observed that the tendency to form mesophases and their stability range were controlled by various factors, which depend on the salts. Thus, in the case of imidazolium and pyridinium salts, increasing chain length increases the existence range of the mesophase [24,30]. On the other hand, in some salts, it is observed that the melting and clearing temperatures depend on the type of cation or counteranion as well [24,26,30]. Therefore, it is considered of importance to carry out a systematic study of the influence of variables to establish the criteria to achieve the control of the mesomorphism, which should be made from novel ILCs. By fabricating ILCs with systematic variations in the type of cations and anions, and in the chain length, it may be possible to rationalize the role of these parameters in the formation of mesophases as well as the existence range. In this way, we aim to identify the most promising compounds within a new family of ILC salts with optimized properties, such as larger existence range of the mesophases and lower melting and higher clearing temperatures.

**Figure 1:**
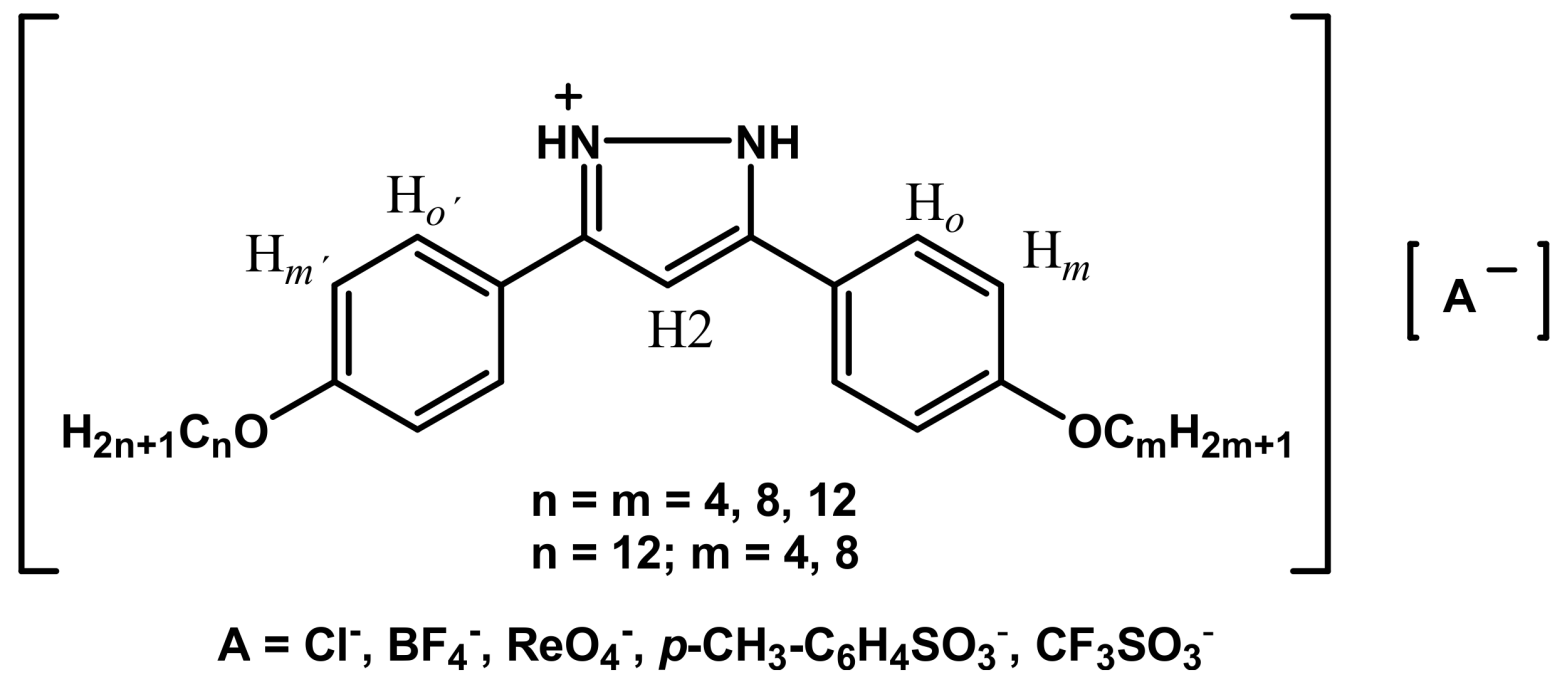
Pyrazolium salts described in this work [H_2_pz^R(n)R(m)^][A].

Recently, we have described a novel type of ILCs based on long-chained alkyloxyphenyl substituted pyrazolium cations [H_2_pz^R(n)^]^+^ which exhibited smectic A (SmA) mesophases when BF_4_^−^, Cl^−^, ReO_4_^−^ and SbF_6_^−^ were used as counteranions, whereas *p*-CH_3_-C_6_H_4_SO_3_^−^ (PTS) and CF_3_SO_3_^−^ (OTf) anions did not lead to LC materials [33]. On this basis, we present herein the study of another new family of ionic salts derived from symmetrically and unsymmetrically substituted pyrazolium cations [H_2_pz^R(n)R(m)^]^+^ (Figure 1) and anions of different type. In particular, we have included two alkyloxyphenyl substituent groups at the 3- and 5-positions of the heterocyclic core of the cation, with alkyl chain lengths of equal or different extension. Furthermore, we have used various anions of different steric and electronic nature. Thus, variables as the length of the substituent chains, the symmetrical and unsymmetrical substitution as well as the steric and electronic effects of the counterions are evaluated on the mesomorphism of compounds. Thus, the focus of this study is to establish the conditions for mesomorphism in these novel ILCs in terms of the mesophase existence range defined by the melting and clearing temperatures. Furthermore, we investigate the dielectric properties of these ILC salts to assess their potential use as ionic conductors [34,35].

Table 1 lists the numbering and nomenclature used for designing the compounds described in this work. For clarity, we use a reduced nomenclature, which includes the anion (or its abbreviation), a dash and the lengths of the two alkyl chains present in the cation, separated by a comma as well as an identification number in parenthesis. For example, the label BF_4_-4,12 refers to the derivative 3-(4-butyloxyphenyl)-5-(4-dodecyloxyphenyl)pyrazolium tetrafluoroborate, [H_2_pz^R(4)R(12)^][BF_4_], which carries the identification number (9).

## 2. Materials and Methods

### 2.1. Materials and Physical Measurements

All commercial reagents were used as supplied. Elemental analysis for carbon, hydrogen, nitrogen and sulfur were carried out by the Microanalytical Service of Complutense University (validated range: C 0.50–94.70%, H 0.50–7.60%, N 0.50–23.00%, S 0.50–30.60%). IR spectra were recorded on a Perkin Elmer Spectrum 100 spectrophotometer (Dpt. Inorganic Chemistry, Complutense University, Madrid, Spain) in the solid state in the 4000–650 cm^−1^ region: vs (very strong), s (strong), m (medium), w (weak), vw (very weak), and b (broad). ^1^H and ^19^F-NMR spectra were performed at room temperature on a Bruker DPX-300 spectrophotometer (300 MHz for ^1^H and 282.40 MHz for ^19^F; NMR Service of Complutense University, Madrid, Spain) from solutions in CDCl_3_. The ^1^H chemical shifts *δ* are listed relative to SiMe_4_ using the signal of the deuterated solvent as reference (7.26 ppm) and coupling constants *J* are in hertz, and they are accurate to ±0.01 ppm and ±0.3 Hz, respectively. Multiplicities are indicated as s (singlet), d (doublet), t (triplet), m (multiplet), and b (broad signal). The ^19^F chemical shifts *δ* are listed relative to trifuorotoluene as external reference and accurate to ±0.1 ppm.

Phase studies were carried out by polarized light optical microscopy (POM) using an Olympus BX50 microscope (Dpt. Inorganic Chemistry, Complutense University, Madrid, Spain) equipped with a Linkam THMS 600 heating stage. The temperatures were assigned based on optic observations with polarized light. Measurements of the transition temperatures were made using a Perkin Elmer Pyris 1 differential scanning calorimeter with the sample (1–4 mg) sealed hermetically in aluminum pans and with a heating or cooling rate of 5–10 K·min^−1^. The X-ray diffractograms at variable temperature were recorded on a Panalytical X’Pert PRO MPD diffractometer with Cu-K*α* (1.54 Å) in a *θ-θ* configuration equipped with an Anton Paar HTK1200 heating stage (X-ray Diffraction Service of Complutense University).

The dielectric properties in powder form were probed by alternating current (AC) impedance spectroscopy using an Alpha Analyzer integrated into the Novocontrol BDS 80 (Dpt. Physics of Materials, Complutense University, Madrid, Spain). Measurements were performed at a frequency (*f*) range of 1 Hz–10 MHz with a 0.1 V applied AC voltage signal, in the temperature (*T*) range of 260 K–480 K (−13 °C–207 °C) upon heating and cooling cycles. Dielectric data were taken under steady state conditions, where the selected *T* was stabilized between 3 and 10 min before taking impedance measurements over the full *f*-range. The temperature increments/reductions for taking impedance measurements were 20 K–2 K steps, where the temperature was increased/decreased in smaller steps near the phase transitions. The compounds in the solid powder state (white/light brown color) were placed between the polished electrodes of a custom-built stainless steel liquid cell with a high surface to thickness ratio [36] as demonstrated in the Supplementary Materials (Figure S1). The cell was closed with a sapphire plate and placed inside the Novocontrol cryostat.

The dielectric data were obtained at selected temperatures for heating and cooling cycles in terms of the real and imaginary parts (*Z′*, *Z″*) of the complex impedance Z* = *Z′* + i*Z″*. The geometrical factor (*g*) is given by the electrode area divided by electrode distance. Due to experimental limitations, *g* could only be estimated from the weight and density of the powder measured initially, and the measurement cell dimensions. Equivalent circuit fitting of the dielectric data was performed by using commercial Z-View software. The conductivity and permittivity values extracted from the equivalent circuit fits were plotted vs. temperature, but only physically meaningful values with sufficiently low fitting errors were considered.

**X-ray data collection and structure refinement:** Data collection was carried out at room temperature on a Bruker Smart CCD diffractometer (X-Ray Diffraction Service of Complutense University, Madrid, Spain) using graphite-monochromated Mo-K*α* radiation (*λ* = 0.71073 Å) operating at 50 kV and 35 mA. The data were collected over a hemisphere of the reciprocal space by combination of three exposure sets. Each exposure of 20 s covered 0.3° in *ω*. The cell parameters were determined and refined by a least-squares fit of all reflections. The first 100 frames were recollected at the end of the data collection to check for possible crystal decay, but no appreciable decay was observed. A semi-empirical absorption correction was applied. A summary of the fundamental crystal and refinement data is given in Table S1.

The structure was resolved by direct methods and refined by full-matrix least-squares procedures on *F*^2^ [37]. All non-hydrogen atoms were refined anisotropically. The chain atoms, O1–C22 and O2–C25, were refined with geometrical restraints and variable common oxygen-carbon and carbon-carbon distances.

The hydrogen atoms bonded to nitrogen and oxygen were located on a difference Fourier map and refined riding on their respective bonded atoms. The remaining hydrogen atoms were included in their calculated positions and refined riding on their respective carbon atom.

CCDC 1823990 contains the supplementary crystallographic data for this work. These data can be obtained free of charge from The Cambridge Crystallographic Data Center via www.ccdc.cam.ac.uk/data_request/cif.

### 2.2. Preparation of 3-(4-Alkyloxyphenyl)-5-(4-alkyloxyphenyl)pyrazole

Compounds Hpz^R(n)R(m)^ (n = m) used as precursors in this work were synthesized according to the procedure previously reported by us [3738]. The related asymmetric compounds Hpz^R(n)R(m)^ (n ≠ m) were obtained by a similar procedure (see Supplementary Materials).

### 2.3. Preparation of 3-(4-Alkyloxyphenyl)-5-(4-alkyloxyphenyl)pyrazolium Salts of the Type Cl-n,m (n = m = 4, 8, 12; n = 4, 8 and m =12) and BF_4_-n,m (n = m = 4, 8, 12; n = 4, 8 and m =12)

To a solution of 3 mmol of the corresponding [Hpz^R(n)R(m)^] in 50 mL of dichloromethane, a hydrochloric or tetrafluoroboric acid solution (HCl spec. grav. 1.19 or HBF_4_·Et_2_O, respectively) was added until a white precipitate was formed. The mixture was stirred at room temperature for 5 h. Then, the solvent was removed up to half volume and cooled to −18 °C (1 h). The solid was filtered, washed with water and dried in vacuum. All compounds were characterized by IR and ^1^H-NMR spectroscopies and CHN analyses (deposited as Supplementary Materials). Specific example of the characterization for each family is given below.

**Cl-4,4 (1):** white solid (65%). Found: C, 66.8; H, 7.0; N, 6.8%. C_23_H_29_N_2_O_2_Cl·0.2CH_2_Cl_2_ requires C, 66.7; H, 7.1; N, 6.7%. ν_max_/cm^−1^: 3230w, ν(NH), 2955m, 2873m ν(CH), 1618vs ν(CC + CN), 830s γ(CH). δ_H_ (300 MHz; CDCl_3_; SiMe_4_): 0.99 (6 H, t, ^3^*J*_H–H_ 7.3, CH_3_), 1.50 (4 H, m, CH_2_), 1.78 (4 H, m, CH_2_), 3.95 (4 H, t, ^3^*J*_H–H_ 6.2, OCH_2_), 5.62 (CH_2_Cl_2_), 6.70 (1 H, b, CH), 6.83 (4 H, d, ^3^*J*_H–H_ 5.1, H*_m_*), 7.65 (4 H, b, H*_o_*).

**BF_4_-4,4 (6):** white solid (36%). Found: C, 59.7; H, 6.2; N, 6.1%. C_23_H_29_N_2_O_2_BF_4_·0.2CH_2_Cl_2_ requires C, 59.9; H, 6.5; N, 6.1%. ν_max_/cm^−1^: 3182m,b ν(NH), 2957m, 2873m ν(CH), 1620vs ν(CC + CN), 1066s ν(BF), 839s γ(CH). δ_H_ (300 MHz; CDCl_3_; SiMe_4_): 0.99 (6 H, t, ^3^*J*_H–H_ 7.3, CH_3_), 1.50 (4 H, m, CH_2_), 1.79 (4 H, m, CH_2_), 4.00 (4 H, t, ^3^*J*_H–H_ 6.4, OCH_2_), 5.62 (CH_2_Cl_2_), 6.85 (1 H, b, CH), 7.01 (4 H, d, ^3^*J*_H–H_ 7.9, H*_m_*), 7.67 (4 H, d, ^3^*J*_H–H_ 8.0, H*_o_*).

### 2.4. Preparation of 3-(4-Alkyloxyphenyl)-5-(4-alkyloxyphenyl)pyrazolium Salts of the Type ReO_4_-n,m (n = m = 4, 8; n = 4, 8 and m =12), PTS-n,m (n = m = 4, 8, 12; n = 4, 8 and m =12) and OTf-n,m (n = m = 4, 8; n = 4, 8 and m =12)

To a solution of 0.5 mmol of the corresponding Cl-n,m compound in 40 mL of dichloromethane, the corresponding salt AgA (A = ReO_4_, OTf, PTS) in 10 mL of a mixture of dichloromethane-acetonitrile (1:3) and in a 1:1 molar ratio was added, under a nitrogen atmosphere. The mixture was stirred for 24 h at room temperature in the absence of light and then filtered through Celite^®^. The filtrate was concentrated in vacuum until a solid precipitated. The white or light brown solid was filtered and dried in vacuum. All compounds were characterized by IR and ^1^H-NMR spectroscopies and CHNS elemental analyses (deposited as Supplementary Materials). A specific example of the characterization for each family is given below.

**ReO_4_-4,4 (11):** light brown solid (37%). Found: C, 45.4; H, 4.7; N, 4.6%. C_23_H_29_N_2_O_2_ReO_4_·0.2CH_3_CN requires C, 45.1; H, 4.8; N, 4.9%. ν_max_/cm^−1^: 3183m ν(NH), 2925m, 2870m ν(CH), 1620s ν(CC + CN), 897vs ν(ReO), 839m γ(CH). δ_H_ (300 MHz; CDCl_3_; SiMe_4_): 0.99 (6 H, t, ^3^*J*_H–H_ 7.1, CH_3_), 1.51 (4 H, m, CH_2_), 1.80 (4 H, m, CH_2_), 4.00 (4 H, t, ^3^*J*_H–H_ 6.3, OCH_2_), 6.85 (1 H, s, CH), 7.02 (4 H, d, ^3^*J*_H–H_ 8.2, H*_m_*), 7.73 (4 H, d, ^3^*J*_H–H_ 8.3, H*_o_*).

**PTS-4,4 (15):** white solid (37%). Found: C, 65.2; H, 6.4; N, 5.5; S, 5.7%. C_30_H_36_N_2_O_5_S·0.2CH_2_Cl_2_ requires C, 65.4; H, 6.8; N, 5.1; S, 5.8%. ν_max_/cm^−1^: 3134m ν(NH), 2959m, 2872m ν(CH), 1621s ν(CC + CN), 1166vs ν_as_(SO_3_), 1035s ν_s_(SO_3_), 827vs γ(CH). δ_H_ (300 MHz; CDCl_3_; SiMe_4_): 0.99 (6 H, t, ^3^*J*_H–H_ 7.4, CH_3_), 1.52 (4 H, m, CH_2_), 1.80 (4 H, m, CH_2_), 2.37 (3 H, s, CH_3_(PTS)), 4.02 (4 H, t, ^3^*J*_H–H_ 6.5, OCH_2_), 5.62 (CH_2_Cl_2_), 6.82 (1 H, s, CH), 7.02 (4 H, d, ^3^*J*_H–H_ 8.7, H*_m_*), 7.22 (2 H, d, ^3^*J*_H–H_ 8.1, H*_o_*(PTS)), 7.73 (4 H, d, ^3^*J*_H–H_ 8.7, H*_o_*), 7.86 (2 H, d, ^3^*J*_H–H_ 8.1, H*_m_*(PTS)).

**OTf-4,4 (20):** light brown solid (43%). Found: C, 56.3; H, 5.6; N, 5.6; S, 5.9%. C_23_H_29_N_2_O_2_CF_3_SO_3_ requires C, 56.0; H, 5.7; N, 5.4; S, 6.2%. ν_max_/cm^−1^: 3150m ν(NH), 2942m, 2874m ν(CH), 1623s ν(CC + CN), 1243vs ν_as_(SO_3_), 1035s ν_s_(SO_3_), 827vs γ(CH). δ_H_ (300 MHz; CDCl_3_; SiMe_4_): 0.99 (6 H, t, ^3^*J*_H–H_ 6.7, CH_3_), 1.52 (4 H, m, CH_2_), 1.81 (4 H, m, CH_2_), 4.04 (4 H, t, ^3^*J*_H–H_ 6.4, OCH_2_), 6.86 (1 H, s, CH), 7.03 (4 H, d, ^3^*J*_H–H_ 8.9, H*_m_*), 7.70 (4 H, d, ^3^*J*_H–H_ 8.7, H*_o_*).

## 3. Results and Discussion

### 3.1. Synthesis and Characterization

Ionic salts containing 3-(4-alkyloxyphenyl)-5-(4-alkyloxyphenyl)pyrazolium cations [H_2_pz^R(n)R(m)^]^+^, bearing alkyl chains of the same or different length (n = m = 4, 8, 12; n = 12, m = 4,8), and five different counteranions Cl^−^, BF_4_^−^, ReO_4_^−^, *p*-CH_3_-C_6_H_4_SO_3_^−^ (PTS) and CF_3_SO_3_^−^(OTf) were synthesized, leading to five homologous series (I, II, III, IV, V) of compounds (Figure 1 and Table 1).

**Scheme 1:**
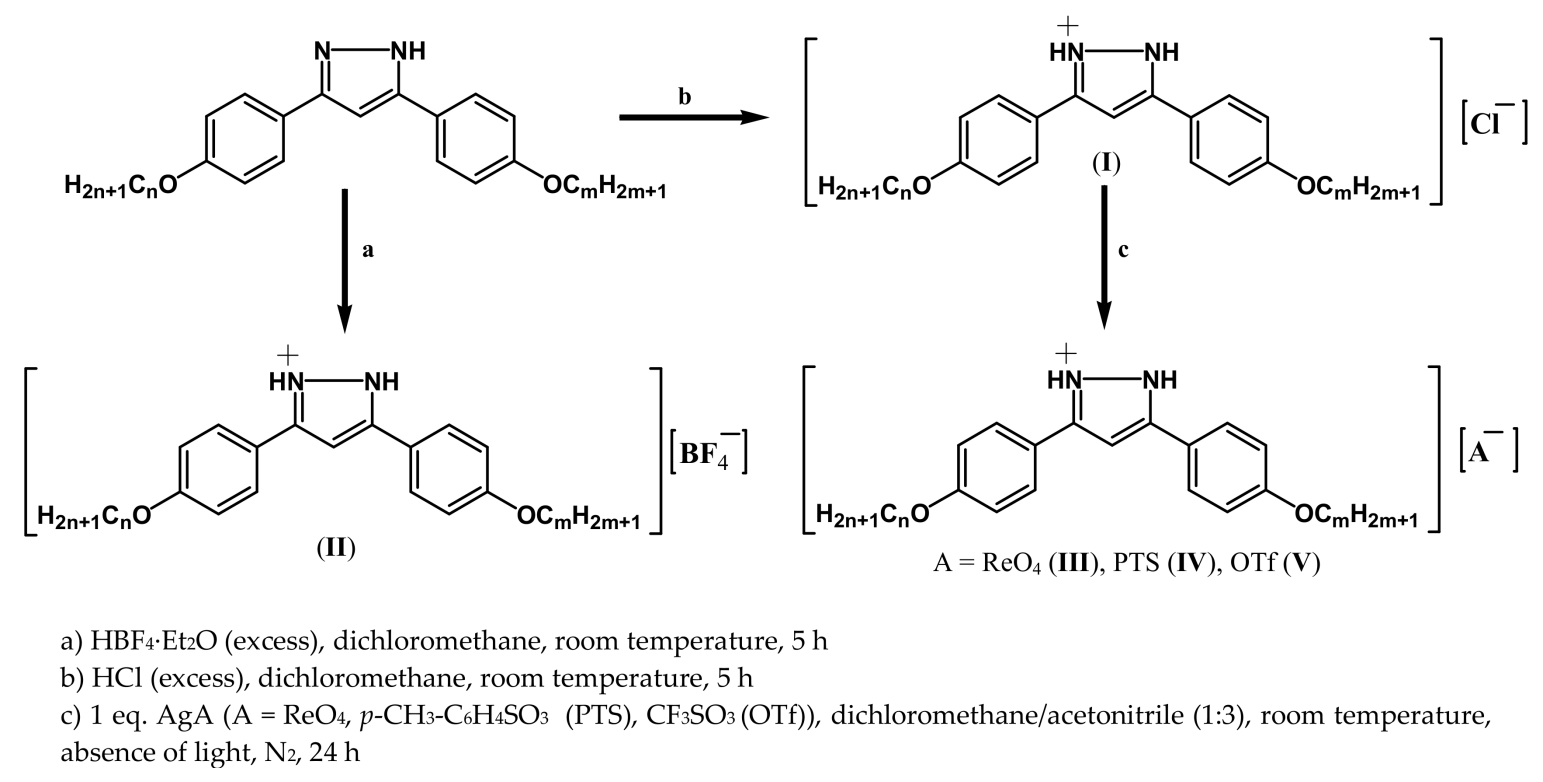
Synthetic route to obtain the ionic salts.

The synthetic procedures of the respective families of salts were dependent on the counteranion (Scheme 1). The Cl-n,m and BF_4_-n,m derivatives were obtained by a direct reaction between the pyrazole and the corresponding acid. The ReO_4_-n,m, OTf-n,m and PTS-n,m derivatives were synthesized by a metathesis reaction from the corresponding Cl-n,m derivatives and the AgA (A = ReO_4_, OTf, PTS) salts in a 1:1 stoichiometric ratio. The final compounds were isolated as white or light brown solid salts, which were stable at room temperature and soluble in common organic solvents. The pyrazole ligands used as precursors of these salts were prepared following the method previously described in the literature [38].

The synthesized salts were fully characterized by elemental analysis and IR, ^1^H-NMR and ^19^F-NMR spectroscopies.

The ^1^H-NMR spectra of the compounds of the series **I**–**V**, recorded in CDCl_3_ solution at room temperature, show well-defined peaks associated with the aromatic and aliphatic protons of the pyrazolium cation (see Section 2 and Supplementary Materials). In the case of the PTS-n,m derivatives, the aromatic proton signals from the anion are observed as well. It is noteworthy that in all cases the expected signals from the protons bound to the N atoms of the pyrazole ring are not observed. The absence of these signals is most likely due to the presence of hydrogen bond interactions involving the NH groups. As a support of this consideration, the X-ray structure and the IR data, commented below, agree with the presence of hydrogen bonds established in the solid state. This tendency of the pyrazolium derivatives to generate NH-hydrogen bonds could also be considered to be produced in solution, being able to implicate both solvent molecules of their own constitution of water from the deuterated solvent.

Interestingly, the spectra for compounds with asymmetric chains do not display the expected duplicate signals, most likely due to the rapid exchange of the NH protons which leads to an intermediate single signal. In all cases, the protonation of the N in the pyrazole ring leads to a downfield shift of the signals with respect to those observed in the precursor pyrazole [38]. This shift is greater for the *ortho* protons of the phenyl ring (0.05–0.37 ppm), which is coherent with their positions closer to the nitrogen atom. This is particularly significant for the Cl derivatives. In the case of the *meta* protons, the chemical shift is markedly lower (0.04–0.12 ppm). Likewise, the signal from the H4 proton of the pyrazole ring also shifts to higher values (between 0.06 and 0.17 ppm). In contrast, the aliphatic signals are not significantly altered by the protonation effect, independently of the counteranion present in the salt. The species containing BF_4_-n,m and OTf-n,m anions exhibit similar chemical shift values independently of the chain lengths and the anion, which could be attributed to the determinant effect produced by the fluorine atom of the respective counteranions in the interactions with the cation.

The ^19^F-NMR spectra of the compounds BF_4_-8,12 (10) and OTf-8,12 (23), selected as representative examples, recorded in CDCl_3_ solution at room temperature, exhibit the chemical shifts of the fluorine atoms in the expected range (Figure S2) [39,40]. In particular, the OTf-8,12 (23) spectrum shows a single peak around −78 ppm, in agreement with the presence of the fluorinated anion as well as the equivalence of the fluorine atoms therein. On the other hand, BF_4_-8,12 (10) displays a signal close to −149 ppm consisting of two very close lines with intensity ratio of 1:4, coinciding with the natural abundance of ^10^B and ^11^B boron isotopes [41]. In both cases, the chemical shifts agree well with those found in other fluorinated ionic salts [33].

Solid state IR spectra of pyrazolium salts (series **I**–**V**) have been recorded in the 4000–650 cm^−1^ region. In all cases, the spectra show similar patterns with characteristic bands for the respective cations and anions (see Experimental Section). The single weak band at 3130–3260 cm^−1^ was assigned to the ν(NH) vibrations, which indicates the equivalence of both NH bands of the cationic pyrazole ring. In a previous work, it has been reported that the ν(NH) values of several pyrazoles depend on the presence or absence of intermolecular H-bonding interactions, which leads to dimers with ν(NH) at 3260 cm^−1^ or monomers at 3420 cm^−1^, respectively [41]. Based on this argument, we consider that in our salts H-bonding interactions may also be likely. The band around 1620 cm^−1^ is assigned to the ν(CN) vibration of the pyrazole ring, which overlaps with that of the ν(CC) vibrations of the aromatic rings. When modifying the alkyl chain length, no significant differences were observed in the vibrational frequencies, although an increase in the intensity of the aliphatic ν(CH) bands was observed by increasing the chain length. The presence of the anions was established through their characteristic bands that were found at similar values to those of related ionic salts [42]. This confirms their inclusion as uncoordinated ions without excluding a potential contribution to hydrogen-bond interactions (see Experimental Section). In particular, the bands at about 1070 and 930 cm^−1^ were assigned to the ν(BF) and ν(ReO) vibrations of the BF_4_^−^ and ReO_4_^−^ anions respectively, while the presence of OTf and PTS anions was confirmed from the two bands at ≈1240 and 1030 cm^−1^, which were associated to the vibrations ν_as_(SO_3_) and ν_s_(SO_3_) respectively.

### 3.2. X-Ray Crystal Structure of [H_2_pz^R(4),R(4)^][ReO_4_]·H_2_O, (ReO_4_-4,4·H_2_O), (11·H_2_O)

The crystalline structure of the representative *ReO_4_-4,4·H_2_O (11·H_2_O)* species was solved by single crystal X-ray diffraction. Suitable crystals were obtained by slow evaporation of a CH_2_Cl_2_/acetone mixture.

**Figure 2:**
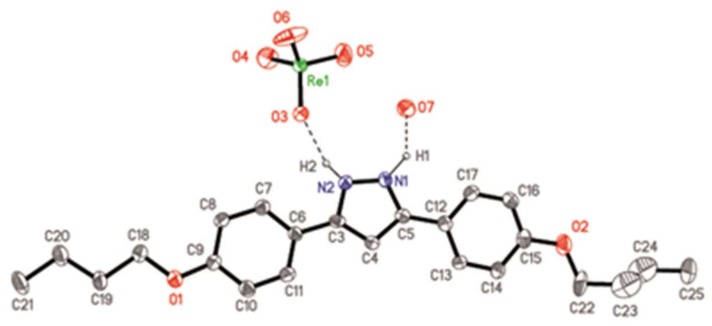
ORTEP representation of ReO_4_-4,4·H_2_O (11·H_2_O) at 20% probability. Hydrogen atoms, except H1 and H2, have been omitted for clarity.

The [H_2_pz^R(4),R(4)^][ReO_4_] compound crystallizes in the space group *C2/c*, where the asymmetric unit contains one crystallographically independent [H_2_pz^R(4)R(4)^]^+^ cation and one ReO_4_^−^ anion (Figure 2), in addition to one molecule of water of crystallization. The distances and bond angles in the pyrazole and phenyl rings of the organic cation are in the expected range (Table S2) and evidence the existence of a delocalized π system, comparable to equivalent molecules described in the literature [33]. One of the phenyl rings (C6–C11) is practically coplanar with the pyrazole ring (dihedral angle: 2.0(1)°), while the second one (C12–C17) exhibits a small tilting angle (dihedral angle: 9.5(1)°). In this situation, the two phenyl rings are rotated 10.4(1)° to each other. The distances and bond angles within the aliphatic chains are consistent with single C–C bonds (d(C–C)_mean_ = 1.52 Å, <(C–C–C)_mean_ = 111.3°). One of the chains (O1–C21) is located in the same plane as the ring it is attached to, while the other (O2–C25) is slightly tilted. The inorganic counteranion, in this case ReO_4_^−^, shows typical Re–O distances and O–Re–O angles consistent with its tetrahedral geometry (Table S1).

**Figure 3:**
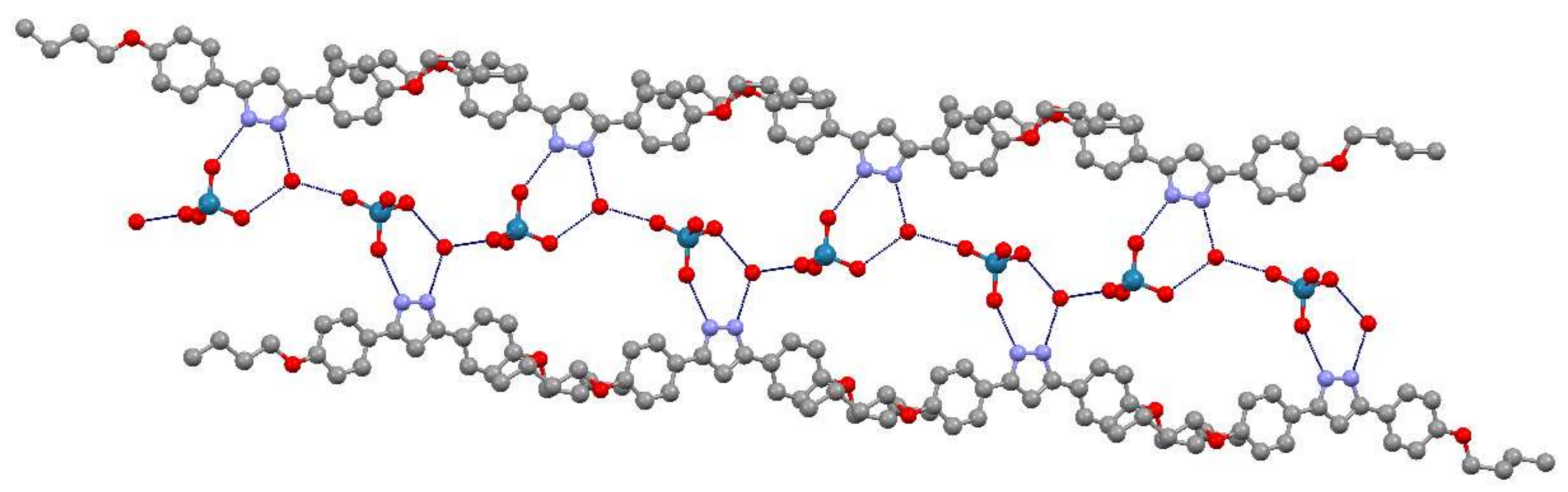
View of the double chain along the *b* axis (hydrogen bonds are represented in blue).

Each single cation (as depicted in Figure 2) is linked to its ReO_4_^−^ anion through a hydrogen bond between the pyrazole N2 atom and the O3 oxygen from the ReO_4_^−^ anion (d(N2···O3) = 2.74(2) Å, <(N2–H2···O3) = 153.1°). The second N1 atom of the pyrazole ring is bound to an oxygen atom from the H_2_O molecule through the hydrogen bond (d(N1···O7) = 2.69(2), <(N1–H1···O7) = 130.4°). Figure 3 demonstrates the connection between cations and anions leading to the formation of chains, where the O7 oxygen of the H_2_O molecule is shown to play a pivotal role by connecting to the O5 oxygen from the counteranion of its own asymmetric unit through a hydrogen bond and additionally to the O4 oxygen from the neighboring counteranion through another hydrogen bond (d(O5···O7) = 2.82(2) Å; d(O4···O7) = 2.73(2) Å, symmetry operation: ½ − *x*, ½ + *y*, ½ − *z*). Thus, the counteranion is involved in three hydrogen bonds connecting three different oxygen atoms (O3, O4 and O5). This fact is manifested in the Re–O distances, where the shortest is related to the oxygen atom that is not included in these connections, i.e., O6 with distance (Re–O6) = 1.59(2) Å, and the remaining distances Re–O are in the interval 1.65(2)–1.69(2) Å. It should be noted that the H_2_O molecule per asymmetric unit is involved in three hydrogen bonds in a trifurcated form. Thus, these hydrogen bonds, facilitated by the H_2_O molecule in each cation–counteranion unit, generate a double chain that propagates along the *b*-axis, where ReO_4_^−^ anions and water molecules are located between organic cations (Figure 3). In this arrangement, the organic cation of one unit is tilted with respect to the cations of the neighboring units by 35.5(1)°.

It is noteworthy that the [H_2_pz^R(4),R(4)^][ReO_4_]·H_2_O (11·H_2_O) salt shows some structural similarities with the monosubstituted pyrazolium salt [H_2_pz^R(1)^][ReO_4_] [33], where in both compounds a one-dimensional supramolecular structure is formed by double strands of stacked anions and cations.

**Figure 4:**
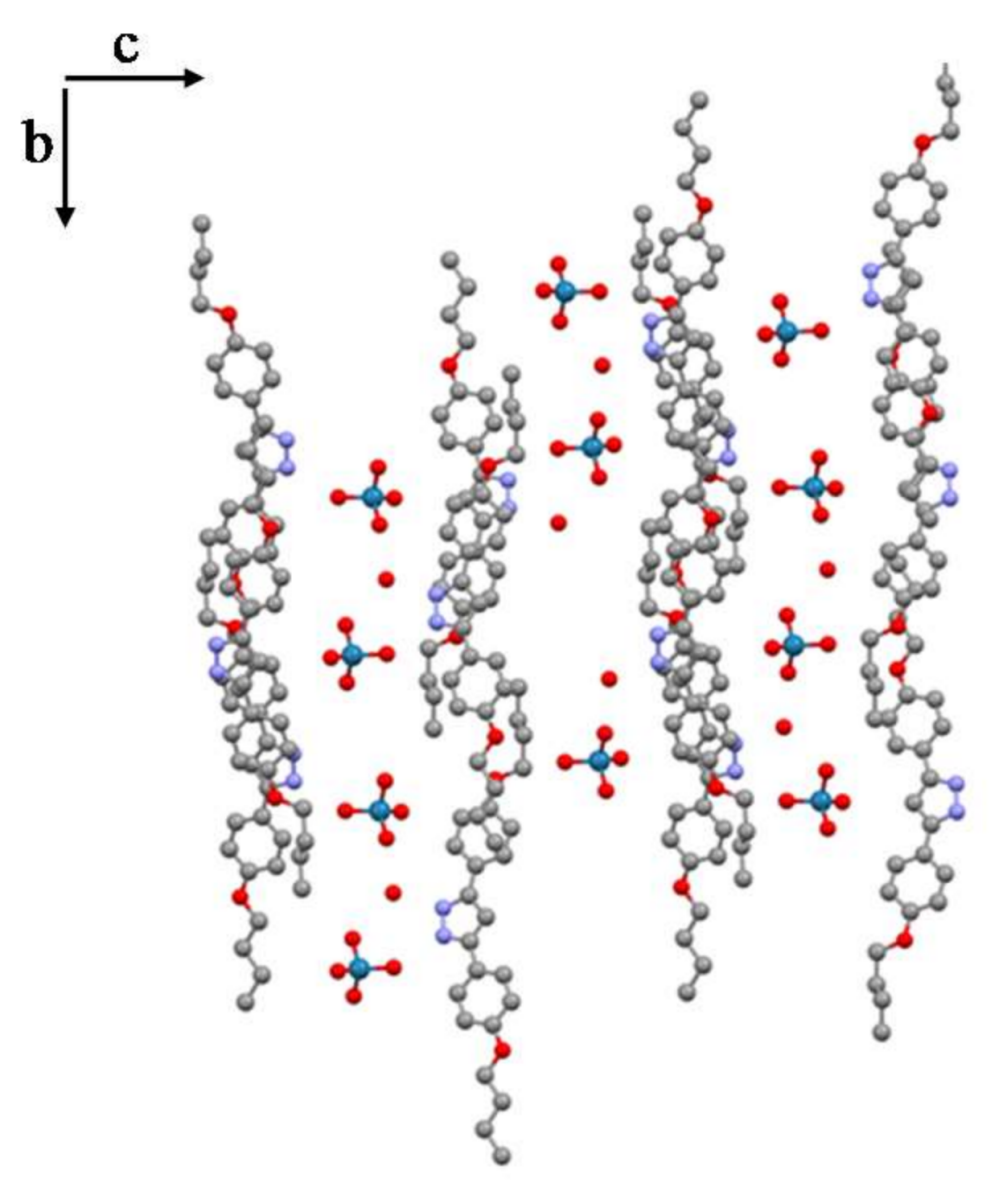
Layers in the *bc* plane showing chain interpenetration.

In the [H_2_pz^R(4),R(4)^][ReO_4_]·H_2_O (11·H_2_O) salt, the presence of a weak interaction by an unconventional weak hydrogen bond C–H···O involving the C4 carbon of the pyrazole ring and the O6 oxygen of a ReO_4_^−^ anion of a different chain (d(C4···O6) = 3.32(2) Å, symmetry operation: *x*, 2 − *y*, *z*), allows the formation of a two-dimensional layered structure, with the layers practically parallel to the *bc* plane. Within each layer a distribution of organic cations with the anions and water molecules located between them is observed and a certain interpenetration of the chains within the cationic part is also formed (Figure 4 and Figure S3).

**Figure 5:**
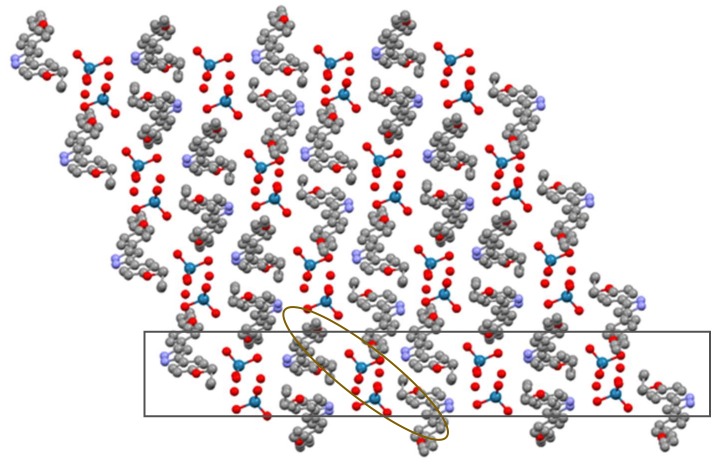
Packing in the *ac plane* (ellipse: double column, rectangle: layer).

Since no further interactions occur, the overall distribution can therefore be described by double chains forming one layer, without subsequent contacts between them (Figure 5).

In summary, it is important to highlight that segregation of hydrophilic and hydrophobic zones occurs, the hydrogen bonds being responsible for the layered crystalline structure.

### 3.3. Thermal Behavior

The thermal behavior of the novel ionic compounds has been examined by polarized light optical microscopy (POM), differential scanning calorimetry (DSC) and X-ray diffraction at low angles and variable temperature. The phase transition temperatures and their corresponding enthalpy values are shown in Table 2 and Table S3.

The pyrazolium salts containing triflate as counteranion (OTf-n,m) did not exhibit LC behavior, but exhibit a rich crystalline polymorphism. POM observations reveal one or more solid–solid transitions between different crystalline polymorphs and then direct fusion to the isotropic liquid (I) from the last solid phase. The DSC thermogram in Figure S4 is consistent with the POM results. The phase transition temperatures together with their enthalpies are shown in Table S3.

**Figure 6:**
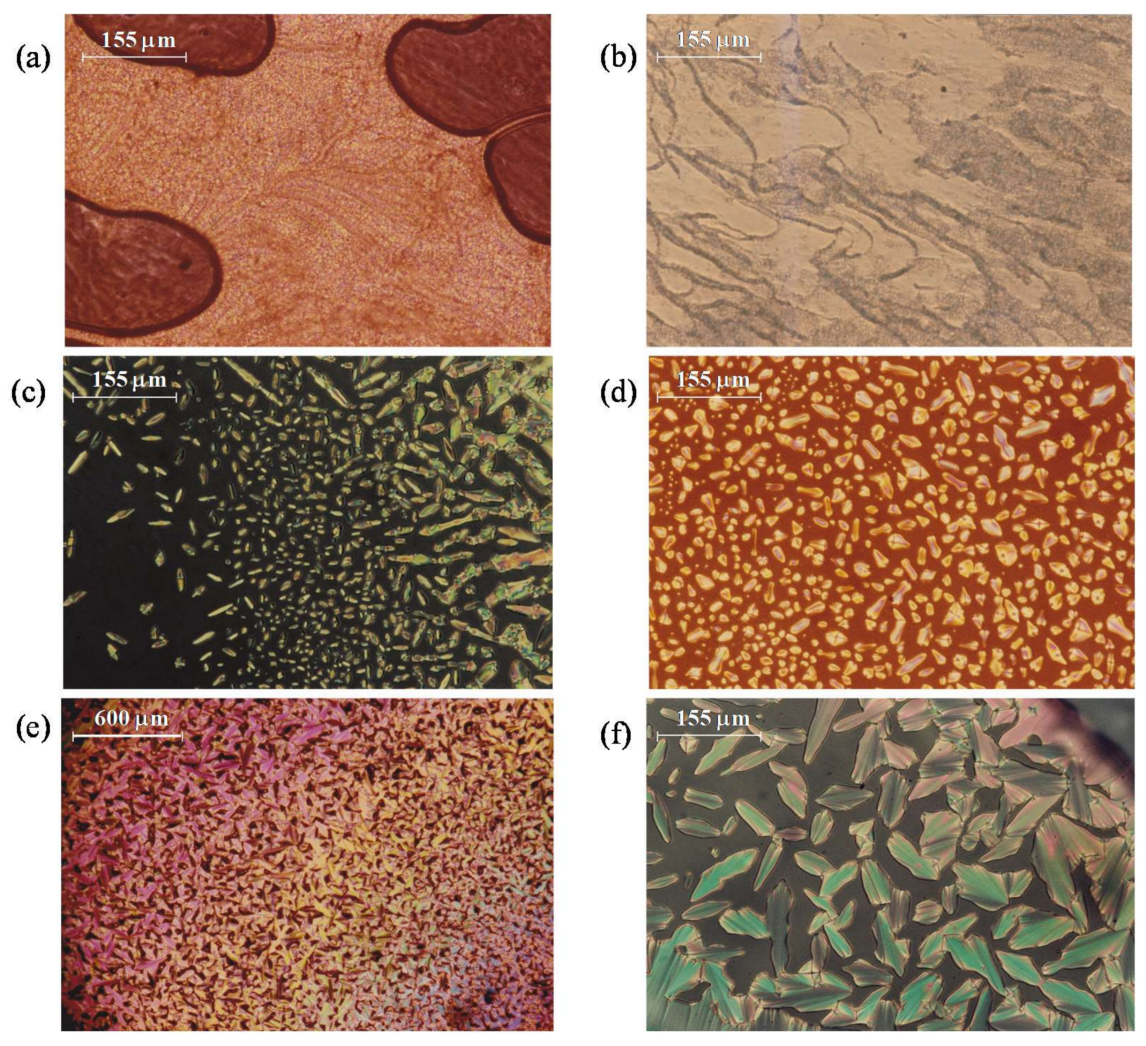
Micrographs with crossed polarizers showing SmA mesophases of compounds: BF_4_-4,12 (9) at 173 °C on heating (**a**); PTS-8,8 (16) at 193 °C on heating (**b**); ReO_4_-4,12 (13) at 227 °C on cooling (**c**); BF_4_-12,12 (8) at 148 °C on cooling (**d**); Cl-4,4 (1) at 191 °C on cooling (**e**); and ReO_4_-4,12 (13) at 208 °C on cooling (**f**).

By contrast, all pyrazolium derivatives containing Cl, BF_4_, ReO_4_ and PTS counteranions exhibit enantiotropic LC behavior, except when the chain length was that with four carbon atoms (6, 11 and 15). In all cases, the mesophases were observed in successive heating/cooling cycles indicating their reversibility, except for the ReO_4_-n,m derivatives. The identification of the mesophases as SmA was achieved by observing through the MOP of an oil-streak texture on heating, when the sample is pressed (Figure 6a,b), and more clearly on cooling from the isotropic liquid by the appearance of *bâtonnets* (Figure 6c,d) which evolve into fan-shaped textures (Figure 6e,f).

The salts containing ReO_4_^−^ counteranions show some decomposition close to the clearing temperatures, which makes it difficult or sometimes impossible to observe the mesophases on cooling. To overcome this inconvenience, POM observations were performed by heating the microscope hot-plate at the clearing temperature and then inserting the sample. Immediately after the sample was cooled, the respective texture could be observed clearly, because decomposition was avoided.

The DSC thermograms measured are consistent with the POM observations and show endothermic peaks, which correspond to the solid–mesophase and mesophase–isotropic liquid transitions on heating, as well as other additional peaks associated with solid–solid transitions in certain derivatives. On cooling, exothermic peaks due to the inverse phase transitions, isotropic liquid–mesophase and mesophase–solid transitions are also observed, except in the cases where decomposition occurred on the heating cycle at high temperatures close to clearing. It is interesting to note that no significant differences in the melting and clearing have been observed between the first and the second heating processes. Figure S5 shows DSC thermograms recorded from one representative example for each family.

The thermograms of the Cl-n,m, BF_4_-n, m and PTS-n,m families show a similar pattern on heating and cooling cycles, with the variations related to the appearance of solid–solid transitions. However, in the case of the ReO_4_-n,m derivatives, the high degree of decomposition observed at temperatures close to the clearing prevents the detection of peaks associated with the mesophase formation and the solidification process on cooling (Figure S4). This mesophase could only be observed by POM as mentioned above. Table 2 displays the thermal data of all mesomorphic salts studied.

**Figure 7:**
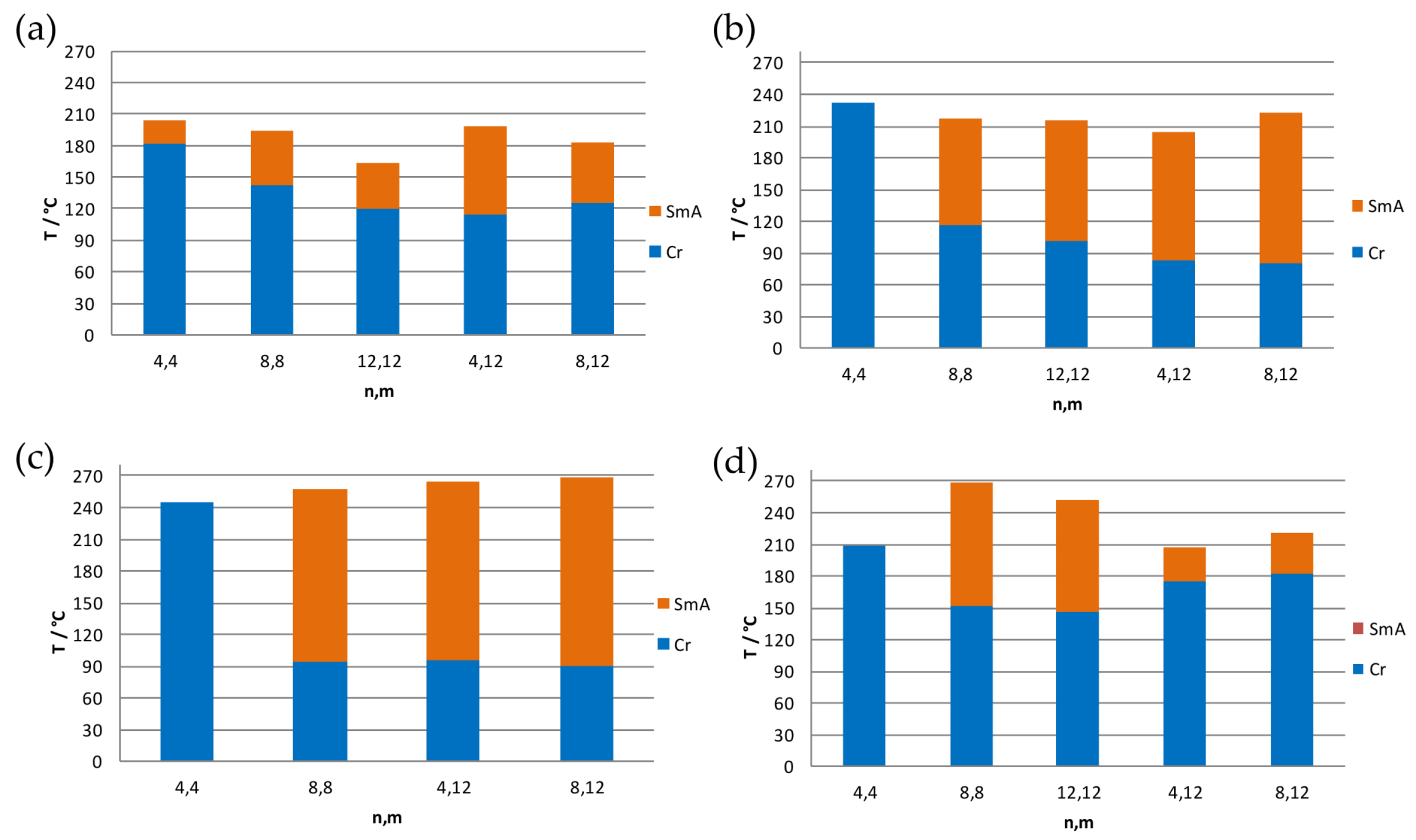
Bar charts of melting and clearing temperatures of the compounds of the following families: Cl-n,m (**a**); BF_4_-n,m (**b**); ReO_4_-n,m (**c**); and PTS-n,m (**d**).

Comparative results of the thermal behavior of all compounds were analyzed to establish the optimized liquid crystal properties. In Figure 7, the bar diagrams indicate the transition temperatures and existence range of the mesophases for all compounds of each family.

In Figure 7, we can consider the influence on the thermal results of two determinant factors: the total length of the alkyl chains (L = n + m) and the asymmetry indicated by the different between the two chains length (Δ = m − n).

Cl-n,m (n = m; n ≠ m): All pyrazolium chloride salts show enantiotropic LC behavior. In the symmetrical derivatives (n = m), the range of existence of the mesophases tendencially increases with chain length, although the melting and clearing temperatures both decrease as expected. In the unsymmetrical species (n ≠ m) containing one chain constant (m = 12), the range of existence increases with increasing asymmetry between the chains, which is manifested by the melting temperature decreasing and the clearing temperature increasing (Figure 7a). It may be concluded that two strategies can be used to improve the mesophase existence range: (a) the increase of the chain length in general; and (b) the increase of asymmetry. From these two strategies, (b) seems to be preferable since the Cl-4,12 (4) compound with the highest degree of asymmetry (L = 16, Δ = 8) exhibits the best LC properties with a large existence range of the mesophase and a relatively high clearing temperature.

BF_4_-n,m (n = m; n ≠ m): The situation is slightly different in the BF_4_-n,m family, where all salts except the BF_4_-4,4 (6) display LC properties. The mesophases appear here above the melting temperatures that are lower than those of their Cl-n,m analogs, which is an advantage. In symmetric species (n = m), an increase in chain length can be associated with a decrease of both the melting and clearing temperatures, similar to the Cl-n,m family. On the other hand, in the asymmetric derivatives with fixed chain length in one of the substituents (m = 12), the increasing asymmetry does not show a clear trend, and the BF_4_-8,12 (10) shows the best LC properties. It may be concluded that the increasing asymmetry somewhat favors the mesomorphism, but the total chain length may be more important here as compared to the Cl-n,m species, thus favoring the BF_4_-8,12 (10) (L = 20, Δ = 4) over the BF_4_-4,12 (9) (L = 16, Δ = 8).

ReO_4_-n,m (n = m; n ≠ m): Equivalent to the BF_4_-n,m derivatives, the ReO_4_-4,4 (11) salt with the shortest chain length does not exhibit mesomorphism. By contrast, the remaining compounds exhibit mesophases, all of them exhibiting similar melting temperatures (90–96 °C) and with the clearing at rather high temperatures in the range of 257–268 °C. The drawback here is the molecular decomposition that occurs near the clearing. Although the species ReO_4_-8,12 (14) (L = 20, Δ = 4) and ReO_4_-4,12 (13) (L = 16, Δ = 8) are similar, again a balance between chain length (L) and asymmetry (Δ) leads to the best LC properties in the ReO_4_-8,12 (14) (Figure 7c), equivalent to the BF_4_-n,m family.

PTS-n,m (n = m; n ≠ m): The compound with the shortest chain length in PTS-4,4 (15) again does not exhibit mesomorphism. The remaining symmetric species show a decrease in the melting and clearing temperature with increasing chain length, equivalent to other families. In contrast, the melting and clearing temperatures in the asymmetric species (m = 12; n = 4, 8) converge with increasing asymmetry (Figure 7d), which is not optimal for designing good LC properties and distinguishes this family to all others.

To summarize, the best LC properties have been observed in the ReO_4_-n,m and BF_4_-n,m families, and in particular in those compounds with the longest total chains length (L = 20). The existence range of the mesophase is more favorable in the ReO_4_-8,12 (14) compound, but decomposition makes the compounds of this family unsuitable for potential application. Consequently, the BF_4_-8,12 (10) compound may constitute the optimum mesomorphic properties suitable for applications.

### 3.4. Variable Temperature Low Angle Powder X-Ray Diffraction Studies

X-ray diffraction experiments were performed at low angles and variable temperature to confirm the identity of the mesophases. This analysis was carried out in one representative compound for each family. Table 3 shows the values obtained for the spacing, Miller indexes and lattice constants.

The X-ray data agree with the deductions and results obtained by POM and DSC. The characteristics of long-range order in the solid phases disappear at the melting temperatures, and in the mesophases only two or three reflections are observed with a ratio of spacing 1:1/2:1/3, which corresponds to (001), (002) and (003) reflections, respectively. A wide *halo* in the region of intermediate angles is associated with the fluid order of the aliphatic chains (Figure S6). These results all confirm the existence of a lamellar mesophase.

By comparing the diffractograms of the Cl-8,8 (2) and Cl-8,12 (5) compounds, we find that increasing chain length increases the interlaminar spacing, which is consistent with greater separation of the layers produced by larger cations. The equivalent tendency is observed in the BF_4_-4,12 (9) and BF_4_-8,12 (10) samples and the PTS-8,8 (16) and PTS-8,12 (19) samples (Table 3).

An interesting result emerges by comparing the interlayer distance of the compounds BF_4_-8,12 (10) and ReO_4_-8,12 (14), containing the same pyrazolium cation and different tetrahedral counteranion. Surprisingly, compound BF_4_-8,12 (10) with the less bulky anion shows a larger interlayer distance than compound ReO_4_-8,12 (14), which has the bulkiest ReO_4_^−^ anion. It is possible to suggest that, in the first case, the potential proximity between layers of cations could be enough to produce higher cation–cation repulsion, thus causing an increased interlayer spacing.

### 3.5. Dielectric Properties

To evaluate possible ionic conductivity in the pyrazolium salts presented, dielectric spectroscopy has been carried out at varying temperatures for representative samples: Cl-8,8 (2), Cl-12,12 (3), BF_4_-8,8 (7), OTf-8,8 (21). The choice of the samples was motivated by the intention to evaluate the influence of the mesophase (the OTf-8,8 shows no mesomorphism), the counteranion form (tetrahedral BF_4_^−^ or spherical Cl^−^), and the chain length for the same counteranion (Cl-8,8 and Cl-12,12).

**Figure 8:**
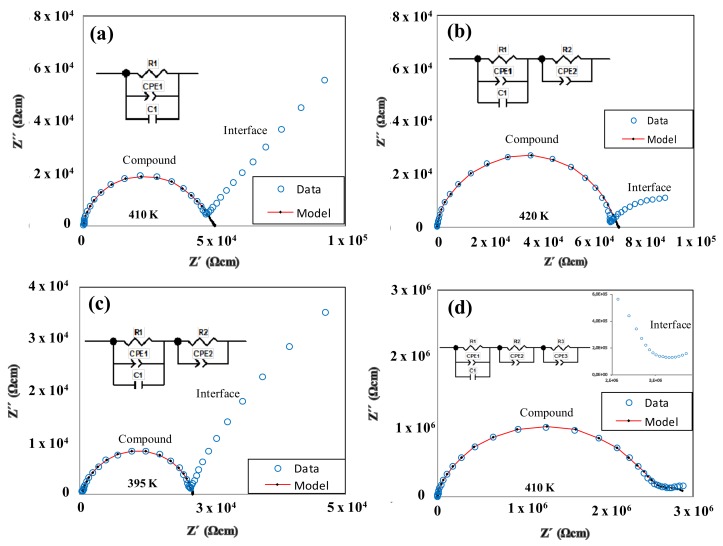
Representations of -*Z″* vs. *Z′* for compounds: BF_4_-8,8 (7) (**a**); Cl-8,8 (2) (**b**); Cl-12,12 (3) (**c**); and OTf-8,8 (21) (**d**).

Figure 8 shows the representations of the real vs. imaginary part of the impedance (−*Z″* vs. *Z′*) at various temperatures within the existence range of the mesophases (BF_4_-8,8, Cl-8,8 and Cl-12,12) or the solid (OTf-8,8).

The main semicircle in each spectrum represents the dielectric contribution of the compound (“Compound”), where the diameter of the semicircle corresponds to the resistivity *ρ* of the charge transport within the compound. The additional semicircle at low frequency (*f*) is an additional dielectric contribution that originates from the interface between the compound and the metallic electrodes (“interface”). For the salt BF_4_-8,8, it is observed that the contribution of the interface has a more linear form in a “pike” shape, which indicates the existence of ionic conductivity unequivocally [43,44,45] (Figure 8a). For Cl-8,8 and Cl-12,12 species, the shape of the interface does not have such a linear “pike” shape, which may well be due to the existence of an electronic contributions to the charge transport or less likely an electrochemical process may occur (Figure 8b,c). Finally, for the derivative OTf-8,8, a second semicircle with a low resistance is observed, which is characteristic of a dominating electronic conduction mechanism (Figure 8d).

**Figure 9:**
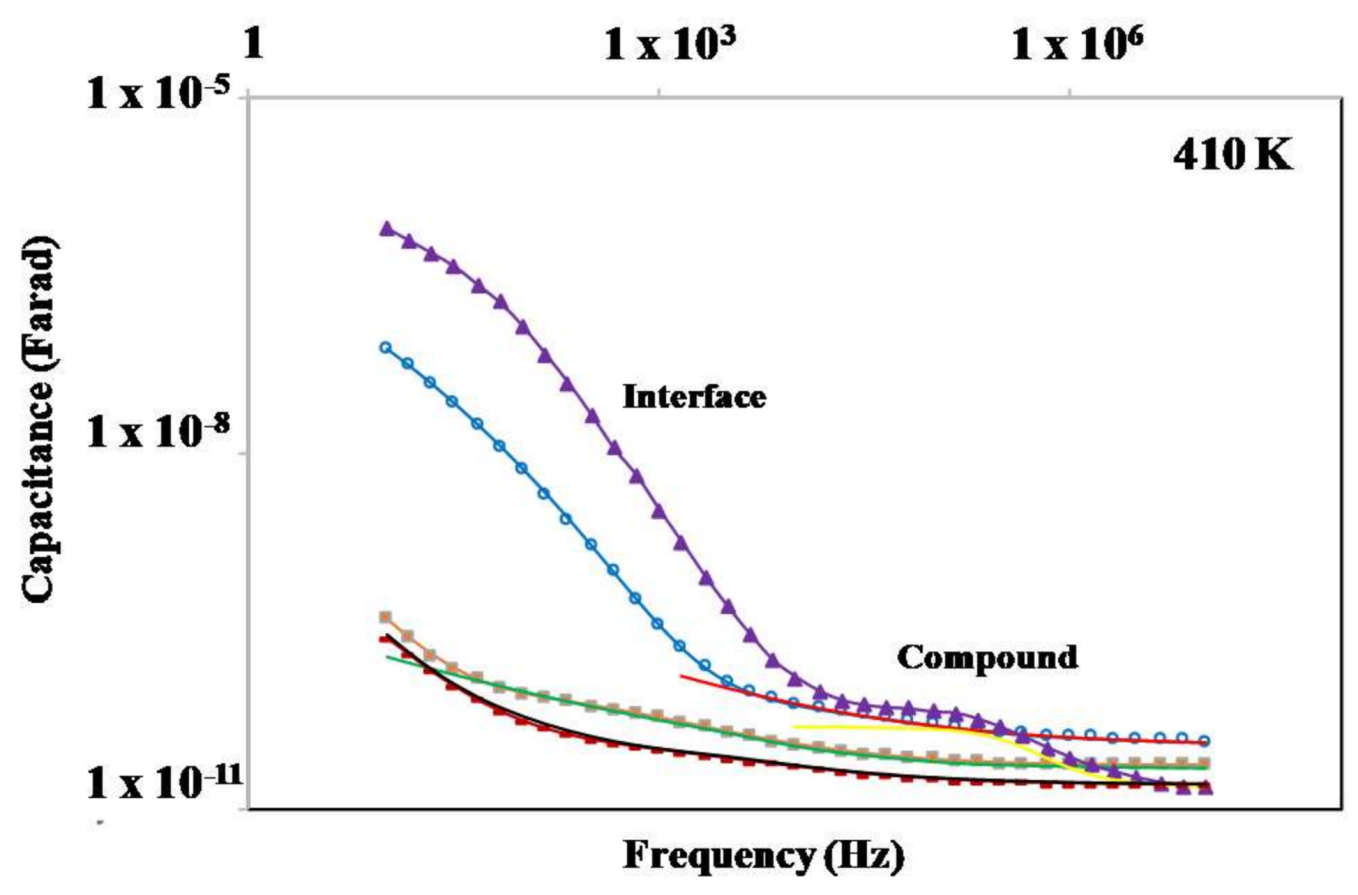
Representation of capacitance *C′* vs. *f* for the studied salts.

Different equivalent circuit models have been used to fit the experimental data for the BF_4_-8,8 (7), Cl-8,8 (2) and Cl-12,12 (3) species at medium and high frequencies where the main semicircle is dominant. In some of the circuit models, the ideal capacitors in the standard RC elements were replaced by a constant phase element (CPE), which reflects the non-ideality of the dielectric contribution. The CPE has been added in parallel to the ideal RC element in some other cases to obtain the most reliable fits possible. In some cases, a second RC element was added to fit the main semicircle, which is justified by a bimodal distribution of the main dielectric contribution as demonstrated below. On the other hand, the two semicircles of compound OTf-8,8 (21) (Figure 8d) were fitted with three R-CPE elements. Indications of three RC elements, including the main contribution with a bimodal distribution, can be observed in the data using the representation of the real part of capacitance *C′* vs. *f*. In the *f*-range where the main (“Compound”) contribution is dominant, the signs of two plateaus for the bimodal main contribution (“Compound”) can be observed, whereas a sharp increase in *C′* at lower *f* can be associated with the interface contribution (Figure 9). The approximately linear increase of *C′* with decreasing *f* is another characteristic of the ionic conductivity, in this case associated with a blocking of the ionic charge carriers at the interface between the compound (ionic charge carriers) and the metallic electrodes (electron charge carriers). The bimodal distribution of the main (“Compound”) contribution can be seen best in the Cl-12,12 (3) sample with clearly two *C′* plateaus displayed.

**Figure 10:**
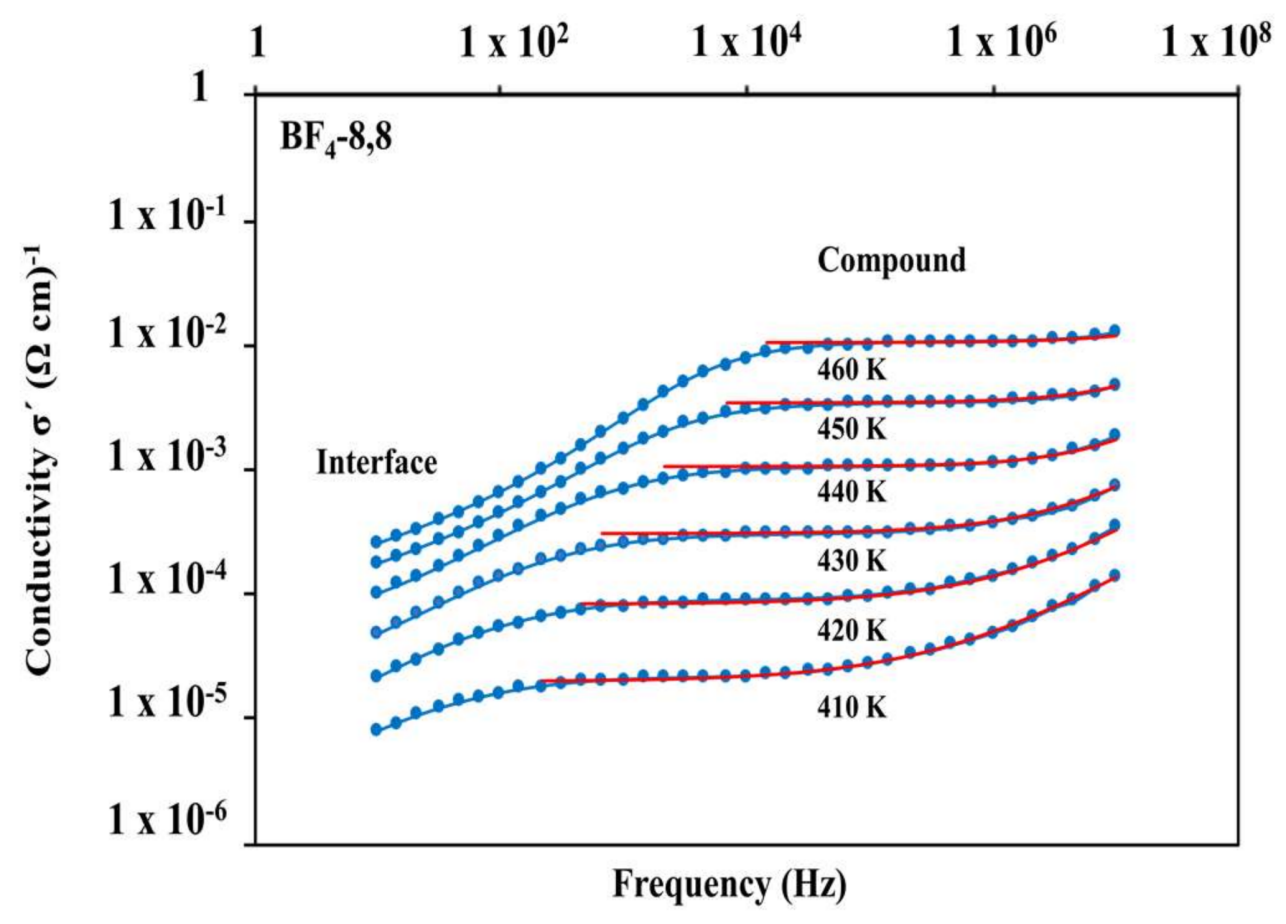
Representation of the real part of the conductivity vs. frequency for the derivative BF_4_-8,8 (7). Blue symbols correspond to the data, while red solid lines represent fits to the data at intermediate and high frequencies, where the main “compound” contribution is dominant.

The real part of the conductivity *σ′* vs. *f* is shown for the representative compound BF_4_-8,8 (7) (Figure 10). The plateau observed at intermediate frequencies (“Compound”) indicates the range of ionic conductivity within the species BF_4_-8,8 (7) at different temperatures. Due to limited data available at high *f*, the possibility of an increase of *σ′* with increasing *f* cannot be ruled out, which would be the typical dielectric response of the Jonscher type at high *f*. Especially at lower temperatures, the upturn of the conductivity at high *f* suggests the existence of this phenomenon.

**Figure 11:**
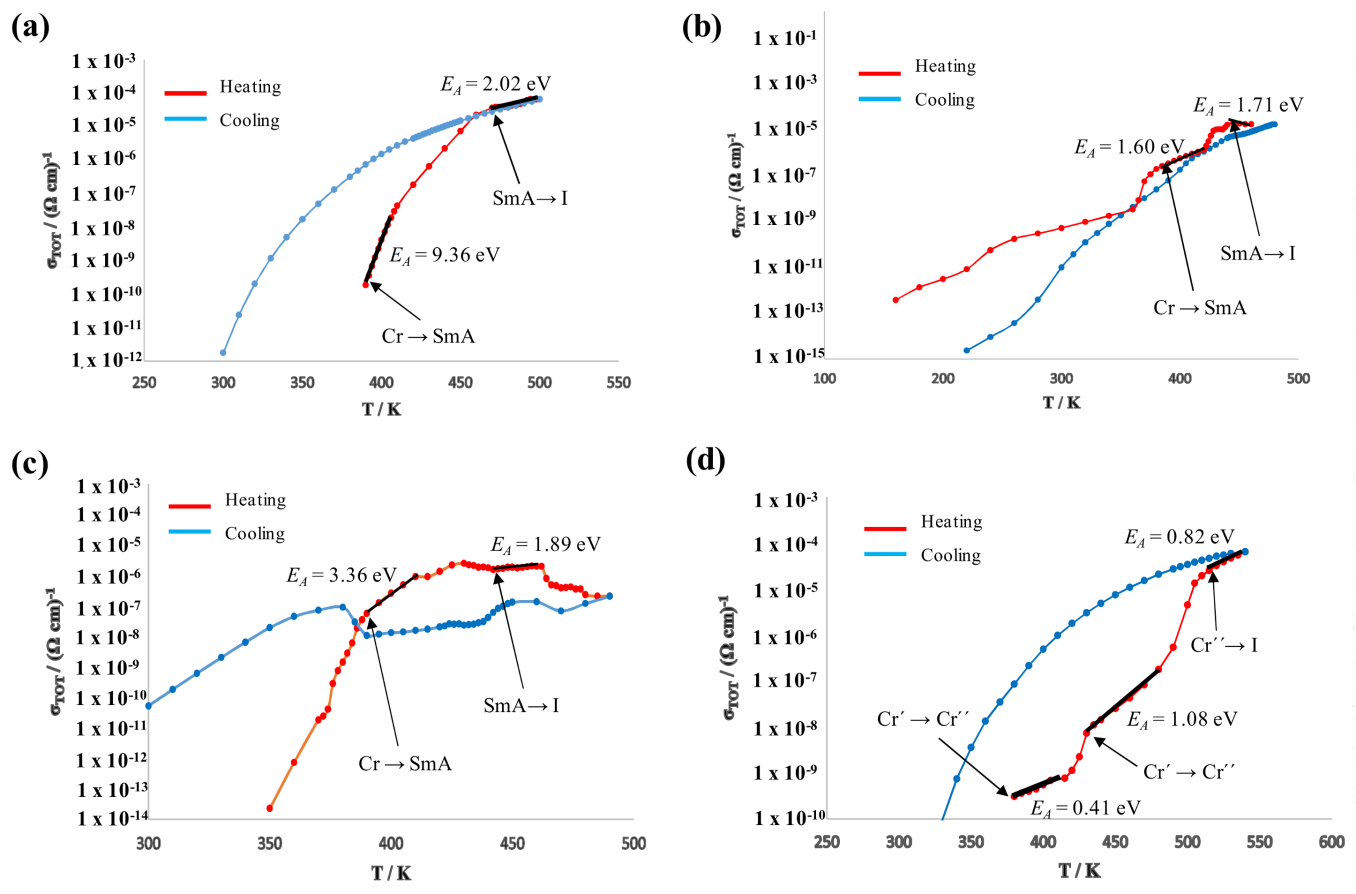
Representations of *σ′* vs. *T* for the salts: BF_4_-8,8 (**a**); Cl-8,8 (**b**); Cl-12,12 (**c**); and OTf-8,8 (**d**).

Figure 11 shows the representations of the conductivity values, extracted from the equivalent circuit models. The experimental data have been collected on heating (red symbols and curves) and cooling (blue), and several phase transitions are reflected by transitional features in the *σ’* vs. *T* curves. From the corresponding ln*σ′* vs. 1/*T* Arrhenius plots (not shown), the activation energies (*E*_A_) of the observed phases were calculated (Table 4) in the temperature ranges where the Arrhenius plots showed good linearity.

The *E*_A_ values calculated in each of the mesophases are fairly similar in the range of 2 eV, which are rather high values that would be consistent with the presence of ionic conductivity (Table 4). Since the first three compounds investigated (BF_4_-8,8, Cl-8,8, Cl-12,12) possess quite different levels of solvents (0.4CH_2_Cl_2_, 0.1CH_2_Cl_2_ and no solvent, respectively), the conductivity in the mesophase may not be influenced significantly by the solvent. However, there is a large difference between the *E*_A_ values detected in the solid phases of the salts investigated. It should be noted at this point that the typical ionic conductivity of ionic liquids (ILs) is associated with a long range mobility of the entire cationic and anionic molecular components, which would be consistent with high *E*_A_ values in the range of 2 eV. It is reasonable to assume that perceptible mobility of the entire ionic components, most likely the counteranion, may occur in the liquid phase or mesophase only, but not in the solid phase. Therefore, the conduction mechanism in the solid phase can be expected to be electronic in nature or, less likely, by the mobility of a different ionic specie. Especially the high *E*_A_ value in the BF_4_-8,8 (7) solid phase is indicative of good electrically insulating behavior, which would be consistent with a dominating ionic conductivity with a considerably lower activation energy in the mesophase. On the other hand, the lower *E*_A_ values in the solid phase of all other compositions may be consistent with a perceptible electronic contribution to the charge transport in the solid and mesophase, which thus inhibits pure ionic conductivity in the latter as argued above. In the Cl-8,8 (2) derivative, the *E*_A_ values in the solid and mesophase are similar, which points to a strong electronic contribution as manifested by two semicircles in Figure 11b. On the other hand, the Cl-12,12 (3) derivative shows a slightly higher *E*_A_ in the solid phase as compared to the mesophase, where ionic conduction may be more dominating as compared to Cl-8,8 (2), and the interface contribution is thus more “pike” (Figure 11c) in the Cl-12,12 (3). The conductivities of the main “Compound” contributions at 460 K are listed for comparison in Table 4.

In summary, only the BF_4_-8,8 (7) compound shows pure ionic conductivity in the mesophase that may be useful for potential applications. Due to the severe qualitative differences in the conduction mechanisms detected, quantitative comparison of the conductivities may be physically less meaningful.

## 4. Conclusions

Ionic salts [H_2_pz^R(n)R(m)^][A] based on 3,5-alkyloxyphenyl substituted pyrazolium cations and different anions, Cl^−^ (**I**), BF_4_^−^ (**II**), ReO_4_^−^ (**III**), PTS (**IV**) and OTf (**V**), bearing various alkyl symmetrical (n = m) or unsymmetrical (n ≠ m) chain lengths, behave as liquid crystal. Exceptions to this behavior are observed for the salts bearing four carbon atoms at the chains (BF_4_-4,4 (6), ReO_4_-4,4 (11) and PTS-4,4 (15)) and those containing OTf as the counteranion. The mesomorphic salts with tetrahedral ReO_4_^−^ and BF_4_^−^ anions have melting temperatures below 100 °C and then turned out to be ILs. Introduction of asymmetry leads to lower melting points. Then, the unsymmetrical BF_4_-8,12 (10) and ReO_4_-8,12 (14) salts exhibited the lowest melting temperatures, but, through a comparative analysis, the BF_4_-8,12 (10) salt is identified as displaying optimum LC behavior.

The crystal structure of a prototype species, ReO_4_-4,4·H_2_O (11·H_2_O), shows a layered distribution of cations and anions. The extrapolation of this structure to the analogs bearing other chain lengths allows establishing the existence of a relationship between the layered structure in the solid state and that of the lamellar SmA mesophase found for these compounds.

The study of the dielectric properties performed at variable temperature reveals that only the BF_4_-8,8 (7) salt may be regarded a pure ionic conductor in the mesophase, which may be useful for potential energy applications.

## Figures and Tables

**Table 1:** Numbering and nomenclature of the compounds [H_2_pz^R(n)R(m)^][A].

Family	I	II	III	IV	V
n,m	Cl-n,m	BF_4_-n,m	ReO_4_-n,m	PTS-n,m	OTf-n,m
	Label (No.)	Label (No.)	Label (No.)	Label (No.)	Label (No.)
**4,4**	Cl-4,4 (1)	BF_4_-4,4 (6)	ReO_4_-4,4 (11)	PTS-4,4 (15)	OTf-4,4 (20)
**8,8**	Cl-8,8 (2)	BF_4_-8,8 (7)	ReO_4_-8,8 (12)	PTS-8,8 (16)	OTf-8,8 (21)
**12,12**	Cl-12,12 (3)	BF_4_-12,12 (8)	-	PTS-12,12 (17)	-
**4,12**	Cl-4,12 (4)	BF_4_-4,12 (9)	ReO_4_-4,12 (13)	PTS-4,12 (18)	OTf-4,12 (22)
**8,12**	Cl-8,12 (5)	BF_4_-8,12 (10)	ReO_4_-8,12 (14)	PTS-8,12 (19)	OTf-8,12 (23)

**Table 2:** Phase transitions of the compounds of the families I-IV determined by DSC and POM.

Comp.	Transition ^a^	T ^b^/°C	ΔH/kJ mol^−1^	Comp.	Transition ^a^	T ^b^/°C	ΔH/kJ mol^−1^
Cl-4,4 (1)	Cr → SmA	181 ^d^	15.4	ReO_4_-4,4 (11)	Cr → Cr′	138	13.0
	SmA → I	200 ^d^	2.8		Cr′ → I	245	9.8
	I → SmA	199 ^d^	−2.5				
	SmA → Cr	171 ^d^	−16.0				
Cl-8,8 (2)	Cr → SmA	143 ^d^	11.8	ReO_4_-8,8 (12)	Cr → SmA	94	39.6
	SmA → I	199 ^d^	6.1		SmA → I	257 ^f^	2.7
	I → SmA	198 ^d^	−6.0		I → SmA	245 ^e^
	SmA → Cr	139 ^d^	−12.6				
Cl-12,12 (3)	Cr → Cr′	67 ^d^	16.4	ReO_4_-4,12 (13)	Cr → Cr′	85 ^c^	30.0 ^g^
	Cr′ → Cr″	92 ^d^	23.4		Cr′→ SmA	96 ^c^	
	Cr″ → SmA	118 ^d^	13.1		SmA → I	265 ^c^	4.0
	SmA → I	174 ^d^	7.3		I → SmA	235 ^e^	
	I → SmA	173 ^d^	−6.9				
	SmA → Cr	122 ^d^	−12.6				
	Cr → Cr′	66 ^d^	−31.0				
Cl-4,12 (4)	Cr → SmA	114 ^c,d^	5.5	ReO_4_-8,12 (14)	Cr → SmA	90 ^c^	39.0
	SmA → I	199 ^c,d^	12.6		SmA → I	268 ^c^	9.4
	I → SmA	182 ^c,d^	−5.4		I → SmA	250 ^e^	
	SmA → Cr	125 ^c,d^	−8.3				
Cl-8,12 (5)	Cr → SmA	126 ^d^	11.8	PTS-4,4 (15)	Cr → Cr′	88	1.2
	SmA → I	184 ^d^	7.0		Cr′ → Cr″	147	3.9
	I → SmA	183 ^d^	−7.0		Cr″ → I	208	26.1
	SmA → Cr	124 ^d^	−11.7		I → Cr	203	−22.0
BF_4_-4,4 (6)	Cr → Cr′	59	13.6	PTS-8,8 (16)	Cr → Cr′	110 ^c^	8.3
	Cr′ → Cr″	154	3.3		Cr′ → Cr″	131 ^c^	1.7
	Cr″ → Cr″′	194	19.0		Cr″ → SmA	151 ^c^	7.2
	Cr″′ → I	232	1.2		SmA → I	269 ^c,f^	27.1
	I → Cr	160	−7.7				
BF_4_-8,8 (7)	Cr → SmA	116	32.4	PTS-12,12 (17)	Cr → Cr′	115	24.6
	SmA → I	217	5.8		Cr′ → SmA	146	15.9
	I → SmA	163	−2.2		SmA → I	252 ^e,f^	
BF_4_-12,12 (8)	Cr → SmA	101	24.7	PTS-4,12 (18)	Cr → Cr′	108	16.2
	SmA → I	215	7.3		Cr′ → Cr″	132	8.3
	I → SmA	211	−5.6		Cr″ → SmA	175	17.9
	SmA → Cr	63	−8.1		SmA → I	207	5.7
					I → SmA	204	−5.1
					SmA → Cr	164	−4.7
					Cr′ → Cr″	139	−8.1
BF_4_-4,12 (9)	Cr → Cr′	70	27.4 ^g^	PTS-8,12 (19)	Cr → Cr′	99	39.5
	Cr′ → SmA	83			Cr′ → Cr″	172	14.1 ^g^
	SmA → I	205	3.7		Cr″→ SmA	182	
	I → SmA	199	−2.7		SmA → I	221	7.4
					I → SmA	220	−6.2
					SmA → Cr	175	−3.7
					Cr′ → Cr″	152	−8.2
BF_4_-8,12 (10)	Cr → Cr′	71	37.5 ^g^				
	Cr′ → SmA	80					
	SmA → I	223	7.6				
	I → SmA	222	−6.1				
	SmA → Cr	112 ^e^					

^a^ Cr, Cr′, Cr″, Cr″′: solid phases; SmA: smectic A mesophase; I: isotropic liquid; ^b^ DSC onset given in the first heating-cooling process; ^c^ DSC peaks given in the first heating-cooling process; ^d^ DSC peaks given in the second heating-cooling cycle; ^e^ Detected by POM; ^f^ Partial decomposition of the compound; ^g^ Overlapped processes.

**Table 3:** X-ray diffraction results for selected compounds from each family.

Compound	T/°C	2θ/°	d_obs_^a^/Å	d_cal_^a^/Å	[hkl] ^b^	Lattice Constant/Å
Cl-8,8 (2)	170 ^c^	3.0	29.0	29.0	(001)	c = 29.2
		6.0	14.7	14.5	(002)	
		17.0	5.2	-	halo	
Cl-8,12 (5)	160 ^c^	2.7	32.1	32.1	(001)	c = 32.5
		5.4	16.4	16.1	(002)	
		17.0	5.2	-	halo	
BF_4_-4,12 (9)	100	3.6	24.4	24.4	(001)	c = 24.8
		7.2	12.3	12.2	(002)	
		10.4	8.5	8.1	(003)	
		17.0	5.2	-	halo	
BF_4_-8,12 (10)	80	2.6	33.5	33.5	(001)	c = 34.0
		5.1	17.2	16.8	(002)	
		17.0	5.2	-	halo	
ReO_4_-8,12 (14)	110	3.5	25.2	25.2	(001)	c = 25.5
		6.9	12.8	12.6	(002)	
		10.3	8.6	8.4	(003)	
		18.9	4.7	-	halo	
PTS-8,8 (16)	155	4.2	20.8	20.8	(001)	c = 20.9
		8.4	10.5	10.4	(002)	
		12.6	7.0	6.9	(003)	
		17.0	5.2	-	halo	
PTS-8,12 (19)	155	3.3	26.7	26.7	(001)	c = 26.8
		6.6	13.3	13.3	(002)	
		17.0	5.2	-	halo	

^a^ d_obs_ and d_cal_ are observed and calculated diffraction spacing; ^b^ [hkl] are the Miller indices of the reflections; ^c^ Second heating cycle

**Table 4:** Activation energies for compounds BF_4_-8,8 (7), Cl-8,8 (2), Cl-12,12 (3) and OTf-8,8 (21) in the solid state and in the mesophase. Conductivity values measured at 460 K.

Compound	*E*_A_ (Solid) (eV)	*E*_A_ (Mesophase) (eV)	σ_460K_ (Ωcm)^−1^
BF_4_-8,8 (7)	9.36	2.02	2.2 × 10^−5^
Cl-8,8 (2)	1.60	1.71	7.0 × 10^−6^
Cl-12,12 (3)	3.36	1.89	1.9 × 10^−6^
OTf-8,8 (21)	1.08	-	4.3 × 10^−8^

## Supplementary Materials

The following are available online at http://www.mdpi.com/1996-1944/11/4/548/s1, Characterization of Compounds [H_2_pz^R(n)R(m)^][A] (A = Cl^−^, BF_4_^−^, ReO_4_^−^, PTS, OTf); Figure S1: Custom-built liquid-solid measurement cell to obtain the conductivity and dielectric properties by impedance spectroscopy in the powder and liquid-crystalline state between the top and bottom electrodes; Figure S2: ^19^F-NMR spectra of compounds BF_4_-8,12 (10) and OTf-8,12 (23) in CDCl_3_ solution at room temperature; Figure S3: Packing of ReO_4_-4,4·H_2_O (11·H_2_O): view of the layer in the *ac* plane (the double columns are marked by brown circles); Figure S4: DSC thermogram of the OTf-4,12 specie (22); Figure S5: DSC thermograms of species Cl-8,8 (2) (a), ReO_4_-8,8 (12) (b), BF_4_-8,8 (7) (c) and PTS-4,12 (18) (d); Figure S6: Diffractogram of compound PTS-8,8 (16) at 155 °C on heating; Table S1: Crystal and refinement data for ReO_4_-4,4·H_2_O (11·H_2_O); Table S2: Distances (Å) and bond angles (°) selected from the compound (ReO_4_-4,4·H_2_O) (11·H_2_O); Table S3: Phase transitions for the salts [H_2_pz^R(n)R(m)^] [OTf] (20 – 23) determined by DSC.

## Abbreviations

The following abbreviations are used in this manuscript:

IL	Ionic Liquid
ILC	Ionic Liquid Crystal
IR	Infrared
LC	Liquid Crystal
LCD	Liquid Crystal Display
I	Isotropic Liquid
Cr, Cr′, Cr″	Solid Phases
Sm	Smectic Mesophase
POM	Polarized light Optical Microscopy
DSC	Differential Scanning Calorimetry
XRD	X-ray Diffraction
CPE	Constant Phase Element
RC	Resistor-Capacitor
NMR	Nuclear Magnetic Resonance
