# Supplementary material for: New Pyrazolium Salts as a Support for Ionic Liquid Crystals and Ionic Conductors
Source: Materials (Basel). 2018 Apr 3;11(4):548. doi: 10.3390/ma11040548 (PMC5951432; doi:10.3390/ma11040548)
Supplement: Supplementary file 1 [file materials-11-00548-s001.pdf]

Supplementary Material: New pyrazolium salts as a support as ionic liquid crystals and ionic conductors

María Jesús Pastor, Ignacio Sánchez, José A. Campo, Rainer Schmidt and Mercedes Cano

Characterization of Compounds [$H_2pz^{R(n)R(m)}$][A] (A = Cl^-, BF_4^-, ReO_4^-, PTS, OTf) by Elemental Analysis (CHN or CHNS), IR, ^{1}H-NMR and, in some cases, ^{19}F-NMR spectroscopies.

Cl-4,4 (1): white solid (65%). Found: C, 66.8; H, 7.0; N, 6.8%. $C_{23}H_{29}N_2O_2Cl{\cdot}0.2CH_2Cl_2$ requires C, 66.7; H, 7.1; N, 6.7%. ν_{max}/cm^{-1}: 3230w ν(NH), 2955m, 2873m ν(CH), 1618vs ν(CC + CN), 830s γ(CH). δ_H (300 MHz; $CDCl_3$; $SiMe_4$): 0.99 (6 H, t, $^3J_{H\text{–}H}$ 7.3, CH_3), 1.50 (4 H, m, CH_2), 1.78 (4 H, m, CH_2), 3.95 (4 H, t, $^3J_{H\text{–}H}$ 6.2, OCH_2), 5.62 (CH_2Cl_2), 6.70 (1 H, b, CH), 6.83 (4 H, d, $^3J_{H\text{–}H}$ 5.1, H_m), 7.65 (4 H, b, H_o).

Cl-8,8 (2): white solid (72%). Found: C, 71.2; H, 8.2; N, 5.4%. $C_{31}H_{45}N_2O_2Cl{\cdot}0.1CH_2Cl_2$ requires C, 71.6; H, 8.7; N, 5.4%. ν_{max}/cm^{-1}: 3210w ν(NH), 2921m, 2851m ν(CH), 1615s ν(CC + CN), 832vs γ(CH). δ_H (300 MHz; $CDCl_3$; $SiMe_4$): 0.88 (6 H, t, $^3J_{H\text{–}H}$ 7.1, CH_3), 1.30 (20 H, m, CH_2), 1.81 (4 H, m, CH_2), 4.01 (4 H, t, $^3J_{H\text{–}H}$ 6.5, OCH_2), 5.62 (CH_2Cl_2), 6.81 (1 H, s, CH), 7.03 (4 H, d, $^3J_{H\text{–}H}$ 8.3, H_m), 8.00 (4 H, d, $^3J_{H\text{–}H}$ 8.5, H_o).

Cl-12,12 (3): white solid (80%). Found: C, 74.8; H, 9.5; N, 4.5%. $C_{39}H_{61}N_2O_2Cl$ requires C, 74.9; H, 9.8; N, 4.5%. ν_{max}/cm^{-1}: 3234w ν(NH), 2922vs, 2850s ν(CH), 1618s ν(CC + CN), 834m γ(CH). δ_H (300 MHz; $CDCl_3$; $SiMe_4$): 0.88 (6 H, t, $^3J_{H\text{–}H}$ 6.9, CH_3), 1.29 (36 H, m, CH_2), 1.81 (4 H, m, CH_2), 4.00 (4 H, t, $^3J_{H\text{–}H}$ 6.6, OCH_2), 6.80 (1 H, s, CH), 7.03 (4 H, d, $^3J_{H\text{–}H}$ 7.9, H_m), 8.01 (4 H, d, $^3J_{H\text{–}H}$ 8.2, H_o).

Cl-4,12 (4): white solid (77%). Found: C, 72.5; H, 8.6; N, 5.4%. $C_{31}H_{45}N_2O_2Cl$ requires C, 72.6; H, 8.8; N, 5.5%. ν_{max}/cm^{-1}: 3202w ν(NH), 2924vs, 2852s ν(CH), 1618vs ν(CC + CN), 837s γ(CH). δ_H (300 MHz; $CDCl_3$; $SiMe_4$): 0.87 (6 H, t, $^3J_{H\text{–}H}$ 6.9, CH_3), 1.30 (20 H, m, CH_2), 1.80 (4 H, m, CH_2), 4.01 (4 H, t, $^3J_{H\text{–}H}$ 6.4, OCH_2), 6.81 (1 H, s, CH), 7.02 (4 H, d, $^3J_{H\text{–}H}$ 8.1, H_m), 8.01 (4 H, d, $^3J_{H\text{–}H}$ 8.3, H_o).

Cl-8,12 (5): white solid (81%). Found: C, 73.1; H, 9.0; N, 4.9%. $C_{35}H_{53}N_2O_2Cl{\cdot}0.1CH_2Cl_2$ requires C, 73.0; H, 9.3; N, 4.8%. ν_{max}/cm^{-1}: 3230w ν(NH), 2922vs, 2850vs ν(CH), 1617vs ν(CC + CN), 837s γ(CH). δ_H (300 MHz; $CDCl_3$; $SiMe_4$): 0.87 (6 H, t, $^3J_{H\text{–}H}$ 7.0, CH_3), 1.28 (28 H, m, CH_2), 1.80 (4 H, m, CH_2), 4.00 (4 H, t, $^3J_{H\text{–}H}$ 6.5, OCH_2), 5.62 (CH_2Cl_2), 6.81 (1 H, s, CH), 7.02 (4 H, d, $^3J_{H\text{–}H}$ 8.1, H_m), 8.02 (4 H, d, $^3J_{H\text{–}H}$ 8.2, H_o).

BF_4-4,4 (6): white solid (36%). Found: C, 59.7; H, 6.2; N, 6.1%. $C_{23}H_{29}N_2O_2BF_4\cdot0.2CH_2Cl_2$ requires C, 59.4; H, 6.3; N, 6.0%. ν_{max}/cm^{-1}: 3182m,b ν(NH), 2957m, 2873m ν(CH), 1620vs ν(CC + CN), 1066s ν(BF), 839s γ(CH). δ_H (300 MHz; $CDCl_3$; $SiMe_4$): 0.99 (6 H, t, $^3J_{H-H}$ 7.3, CH_3), 1.50 (4 H, m, CH_2), 1.79 (4 H, m, CH_2), 4.00 (4 H, t, $^3J_{H-H}$ 6.4, OCH_2), 5.62 (CH_2Cl_2), 6.85 (1 H, b, CH), 7.01 (4 H, d, $^3J_{H-H}$ 7.9, H_m), 7.67 (4 H, d, $^3J_{H-H}$ 8.0, H_o).

BF_4-8,8 (7): white solid (39%). Found: C, 62.9; H, 7.5; N, 4.8%. $C_{31}H_{45}N_2O_2BF_4\cdot0.4CH_2Cl_2$ requires C, 63.0; H, 7.7; N, 4.7%. ν_{max}/cm^{-1}: 3160m,b ν(NH), 2923s, 2853s ν(CH), 1617vs ν(CC + CN), 1065s ν(BF), 834m γ(CH). δ_H (300 MHz; $CDCl_3$; $SiMe_4$): 0.88 (6 H, t, $^3J_{H-H}$ 6.9, CH_3), 1.30 (20 H, m, CH_2), 1.79 (4 H, m, CH_2), 3.98 (4 H, t, $^3J_{H-H}$ 6.5, OCH_2), 5.62 (CH_2Cl_2), 6.84 (1 H, s, CH), 7.00 (4 H, d, $^3J_{H-H}$ 8.3, H_m), 7.66 (4 H, d, $^3J_{H-H}$ 8.4, H_o).

BF_4-12,12 (8): white solid (40%). Found: C, 69.0; H, 8.7; N, 3.8%. $C_{39}H_{61}N_2O_2BF_4$ requires C, 69.2; H, 9.1; N, 4.1%. ν_{max}/cm^{-1}: 3178m,b ν(NH), 2950vs, 2867s ν(CH), 1619vs ν(CC + CN), 1068s ν(BF), 840s γ(CH). δ_H (300 MHz; $CDCl_3$; $SiMe_4$): 0.88 (6 H, t, $^3J_{H-H}$ 7.0, CH_3), 1.28 (36 H, m, CH_2), 1.81 (4 H, m, CH_2), 4.01 (4 H, t, $^3J_{H-H}$ 6.7, OCH_2), 6.80 (1 H, s, CH), 7.02 (4 H, d, $^3J_{H-H}$ 8.0, H_m), 8.01 (4 H, d, $^3J_{H-H}$ 8.1, H_o).

BF_4-4,12 (9): white solid (38%). Found: C, 65.5; H, 7.9; N, 4.9%. $C_{31}H_{45}N_2O_2BF_4$ requires C, 65.9; H, 8.0; N, 5.0%. ν_{max}/cm^{-1}: 3182m,b ν(NH), 2957m, 2873m ν(CH), 1620vs ν(CC + CN), 1066s ν(BF), 839s γ(CH). δ_H (300 MHz; $CDCl_3$; $SiMe_4$): 0.87 (6 H, t, $^3J_{H-H}$ 6.9, CH_3), 1.30 (20 H, m, CH_2), 1.80 (4 H, m, CH_2), 4.01 (4 H, t, $^3J_{H-H}$ 6.4, OCH_2), 6.81 (1 H, s, CH), 7.02 (4 H, d, $^3J_{H-H}$ 8.1, H_m), 8.01 (4 H, d, $^3J_{H-H}$ 8.3, H_o).

BF_4-8,12 (10): white solid (43%). Found: C, 64.5; H, 8.2; N, 4.2%. $C_{35}H_{53}N_2O_2BF_4\cdot1.5H_2O$ requires C, 64.9; H, 8.7; N, 4.3%. ν_{max}/cm^{-1}: 3180m,b ν(NH), 2955m, 2870m ν(CH), 1621vs ν(CC + CN), 1065s ν(BF), 841s γ(CH). δ_H (300 MHz; $CDCl_3$; $SiMe_4$): 0.87 (6 H, t, $^3J_{H-H}$ 6.7, CH_3), 1.29 (28 H, m, CH_2), 1.81 (4 H, m, CH_2), 4.01 (4 H, t, $^3J_{H-H}$ 6.5, OCH_2), 6.81 (1 H, s, CH), 7.03 (4 H, d, $^3J_{H-H}$ 8.1, H_m), 8.01 (4 H, d, $^3J_{H-H}$ 8.4, H_o). δ_F (282.40 MHz; $CDCl_3$): −149.12, −149.18

ReO_4-4,4 (11): light brown solid (37%). Found: C, 45.4; H, 4.7; N, 4.6%. $C_{23}H_{29}N_2O_2ReO_4\cdot0.2CH_3CN$ requires C, 45.1; H, 4.8; N, 4.9%. ν_{max}/cm^{-1}: 3183m ν(NH), 2925m, 2870m ν(CH), 1620s ν(CC + CN), 897vs ν(ReO), 839m γ(CH). δ_H (300 MHz; $CDCl_3$; $SiMe_4$): 0.99 (6 H, t, $^3J_{H-H}$ 7.1, CH_3), 1.51 (4 H, m, CH_2), 1.80 (4 H, m, CH_2), 4.00 (4 H, t, $^3J_{H-H}$ 6.3, OCH_2), 6.85 (1 H, s, CH), 7.02 (4 H, d, $^3J_{H-H}$ 8.2, H_m), 7.73 (4 H, d, $^3J_{H-H}$ 8.3, H_o).

ReO_4-8,8 (12): light brown solid (42%). Found: C, 50.3; H, 6.0; N, 3.8%. $C_{31}H_{45}N_2O_2ReO_4\cdot0.2CH_2Cl_2$ requires C, 50.3; H, 6.1; N, 3.4%. ν_{max}/cm^{-1}: 3134m ν(NH), 2918s,

2870m ν(CH), 1623s ν(CC + CN), 889vs ν(ReO), 823vs γ(CH). δ_H (300 MHz; $CDCl_3$; $SiMe_4$): 0.99 (6 H, t, $^3J_{H-H}$ 7.3, CH_3), 1.50 (20 H, m, CH_2), 1.79 (4 H, m, CH_2), 4.00 (4 H, t, $^3J_{H-H}$ 6.4, OCH_2), 5.62 (CH_2Cl_2), 6.84 (1 H, s, CH), 7.03 (4 H, d, $^3J_{H-H}$ 8.3, H_m), 7.74 (4 H, d, $^3J_{H-H}$ 8.3, H_o).

ReO_4-4,12 (13): light brown solid (40%). Found: C, 51.4; H, 6.0; N, 3.9%. $C_{31}H_{45}N_2O_2ReO_4$ requires C, 51.2; H, 6.2; N, 3.8%. ν_{max}/cm^{-1}: 3136m ν(NH), 2919vs, 2850s ν(CH), 1623vs ν(CC + CN), 890vs ν(ReO), 824s γ(CH). δ_H (300 MHz; $CDCl_3$; $SiMe_4$): 0.87 (6 H, t, $^3J_{H-H}$ 6.7, CH_3), 1.28 (20 H, m, CH_2), 1.80 (4 H, m, CH_2), 4.01 (4 H, t, $^3J_{H-H}$ 6.4, OCH_2), 6.84 (1 H, s, CH), 7.03 (4 H, d, $^3J_{H-H}$ 8.5, H_m), 7.74 (4 H, d, $^3J_{H-H}$ 8.4, H_o).

ReO_4-8,12 (14): light brown solid (39%). Found: C, 54.0; H, 6.6; N, 3.7%. $C_{35}H_{53}N_2O_2ReO_4$ requires C, 53.6; H, 6.8; N, 3.6%. ν_{max}/cm^{-1}: 3134w ν(NH), 2916s, 2850m ν(CH), 1623s ν(CC + CN), 887vs ν(ReO), 822vs γ(CH). δ_H (300 MHz; $CDCl_3$; $SiMe_4$): 0.88 (6 H, t, $^3J_{H-H}$ 6.9, CH_3), 1.27 (28 H, m, CH_2), 1.81 (4 H, m, CH_2), 4.01 (4 H, t, $^3J_{H-H}$ 6.5, OCH_2), 6.85 (1 H, s, CH), 7.03 (4 H, d, $^3J_{H-H}$ 8.7, H_m), 7.75 (4 H, d, $^3J_{H-H}$ 8.7, H_o).

PTS-4,4 (15): white solid (37%). Found: C, 65.2; H, 6.4; N, 5.5; S, 5.7%. $C_{30}H_{36}N_2O_5S{\cdot}0.2CH_2Cl_2$ requires C, 65.4; H, 6.8; N, 5.1; S, 5.8%. ν_{max}/cm^{-1}: 3134m ν(NH), 2959m, 2872m ν(CH), 1621s ν(CC + CN), 1166vs $\nu_{as}(SO_3)$, 1035s $\nu_s(SO_3)$, 827vs γ(CH). δ_H (300 MHz; $CDCl_3$; $SiMe_4$): 0.99 (6 H, t, $^3J_{H-H}$ 7.4, CH_3), 1.52 (4 H, m, CH_2), 1.80 (4 H, m, CH_2), 2.37 (3 H, s, CH_3(PTS)), 4.02 (4 H, t, $^3J_{H-H}$ 6.5, OCH_2), 5.62 (CH_2Cl_2), 6.82 (1 H, s, CH), 7.02 (4 H, d, $^3J_{H-H}$ 8.7, H_m), 7.22 (2 H, d, $^3J_{H-H}$ 8.1, H_o(PTS)), 7.73 (4 H, d, $^3J_{H-H}$ 8.7, H_o), 7.86 (2 H, d, $^3J_{H-H}$ 8.1, H_m(PTS)).

PTS-8,8 (16): white solid (43%). Found: C, 67.4; H, 8.0; N, 4.4; S, 4.9%. $C_{38}H_{52}N_2O_5S{\cdot}0.4CH_2Cl_2$ requires C, 67.5; H, 7.8; N, 4.1; S, 4.7%. ν_{max}/cm^{-1}: 3148m ν(NH), 2918vs, 2853s ν(CH), 1624s ν(CC + CN), 1168vs $\nu_{as}(SO_3)$, 1035m $\nu_s(SO_3)$, 836vs γ(CH). δ_H (300 MHz; $CDCl_3$; $SiMe_4$): 0.89 (6 H, t, $^3J_{H-H}$ 6.6, CH_3), 1.30 (20 H, m, CH_2), 1.77 (4 H, m, CH_2), 2.32 (3 H, s, CH_3(PTS)), 3.95 (4 H, t, $^3J_{H-H}$ 6.5, OCH_2), 5.62 (CH_2Cl_2), 6.74 (1 H, s, CH), 6.94 (4 H, d, $^3J_{H-H}$ 8.2, H_m), 7.21 (2 H, d, $^3J_{H-H}$ 8.1, H_o(PTS)), 7.73 (4 H, d, $^3J_{H-H}$ 8.2, H_o), 7.85 (2 H, d, $^3J_{H-H}$ 8.1, H_m(PTS)).

PTS-12,12 (17): white solid (46%). Found: C, 69.8; H, 8.5; N, 3.7; S, 4.2%. $C_{46}H_{68}N_2O_5S{\cdot}0.5CH_2Cl_2$ requires C, 69.4; H, 8.8; N, 3.5; S, 4.0%. ν_{max}/cm^{-1}: 3140m ν(NH), 2920vs, 2849s ν(CH), 1622s ν(CC + CN), 1170vs $\nu_{as}(SO_3)$, 1034m $\nu_s(SO_3)$, 837vs γ(CH). δ_H (300 MHz; $CDCl_3$; $SiMe_4$): 0.88 (6 H, t, $^3J_{H-H}$ 6.7, CH_3), 1.30 (36 H, m, CH_2), 1.79 (4 H, m, CH_2), 2.34 (3 H, s, CH_3(PTS)), 3.99 (4 H, t, $^3J_{H-H}$ 6.5, OCH_2), 5.62 (CH_2Cl_2), 6.75 (1 H, s, CH), 6.94 (4 H, d, $^3J_{H-H}$ 8.3, H_m), 7.20 (2 H, d, $^3J_{H-H}$ 8.2, H_o(PTS)), 7.73 (4 H, d, $^3J_{H-H}$ 8.3, H_o), 7.85 (2 H, d, $^3J_{H-H}$ 8.2, H_m(PTS)).

PTS-4,12 (18): white solid (38%). Found: C, 70.3; H, 8.1; N, 4.2; S, 4.7%. $C_{38}H_{52}N_2O_5S{\cdot}0.2CH_2Cl_2$ requires C, 69.9; H, 7.9; N, 4.2; S, 4.8%. ν_{max}/cm^{-1}: 3141m ν(NH), 2928vs, 2852s ν(CH), 1622s ν(CC + CN), 1167vs $\nu_{as}(SO_3)$, 1035m $\nu_s(SO_3)$, 836vs γ(CH). δ_H (300 MHz; $CDCl_3$; $SiMe_4$): 0.88 (6 H, t, $^3J_{H-H}$ 6.5, CH_3), 1.29 (20 H, m, CH_2), 1.80 (4 H, m, CH_2), 2.32 (3 H, s, CH_3(PTS)), 4.00 (4 H, t, $^3J_{H-H}$ 6.3, OCH_2), 5.62 (CH_2Cl_2), 6.74 (1 H, s, CH), 6.94 (4 H, d, $^3J_{H-H}$ 8.2, H_m), 7.20 (2 H, d, $^3J_{H-H}$ 8.1, H_o(PTS)), 7.74 (4 H, d, $^3J_{H-H}$ 8.2, H_o), 7.84 (2 H, d, $^3J_{H-H}$ 8.1, H_m(PTS)).

PTS-8,12 (19): white solid (40%). Found: C, 69.9; H, 8.1; N, 4.0; S, 4.4%. $C_{42}H_{60}N_2O_5S{\cdot}0.2CH_2Cl_2$ requires C, 70.2; H, 8.4; N, 3.9; S, 4.4%. ν_{max}/cm^{-1}: 3135m ν(NH), 2933vs, 2849s ν(CH), 1624s ν(CC + CN), 1166vs $\nu_{as}(SO_3)$, 1030m $\nu_s(SO_3)$, 834vs γ(CH). δ_H (300 MHz; $CDCl_3$; $SiMe_4$): 0.88 (6 H, t, $^3J_{H-H}$ 6.3, CH_3), 1.29 (28 H, m, CH_2), 1.81 (4 H, m, CH_2), 2.34 (3 H, s, CH_3(PTS)), 4.00 (4 H, t, $^3J_{H-H}$ 6.1, OCH_2), 5.62 (CH_2Cl_2), 6.75 (1 H, s, CH), 6.94 (4 H, d, $^3J_{H-H}$ 8.2, H_m), 7.19 (2 H, d, $^3J_{H-H}$ 8.1, H_o(PTS)), 7.74 (4 H, d, $^3J_{H-H}$ 8.2, H_o), 7.85 (2 H, d, $^3J_{H-H}$ 8.1, H_m(PTS)).

OTf-4,4 (20): light brown solid (43%). Found: C, 56.3; H, 5.6; N, 5.6; S, 5.9%. $C_{23}H_{29}N_2O_2CF_3SO_3$ requires C, 56.0; H, 5.7; N, 5.4; S, 6.2%. ν_{max}/cm^{-1}: 3150m ν(NH), 2942m, 2874m ν(CH), 1623s ν(CC + CN), 1243vs $\nu_{as}(SO_3)$, 1035s $\nu_s(SO_3)$, 827vs γ(CH). δ_H (300 MHz; $CDCl_3$; $SiMe_4$): 0.99 (6 H, t, $^3J_{H-H}$ 6.7, CH_3), 1.52 (4 H, m, CH_2), 1.81 (4 H, m, CH_2), 4.04 (4 H, t, $^3J_{H-H}$ 6.4, OCH_2), 6.86 (1 H, s, CH), 7.03 (4 H, d, $^3J_{H-H}$ 8.9, H_m), 7.70 (4 H, d, $^3J_{H-H}$ 8.7, H_o).

OTf-8,8 (21): light brown solid (41%). Found: C, 60.4; H, 7.2; N, 4.5; S, 5.4%. $C_{31}H_{45}N_2O_2CF_3SO_3{\cdot}0.2CH_2Cl_2$ requires C, 60.7; H, 7.2; N, 4.4; S, 5.1%. ν_{max}/cm^{-1}: 3160m ν(NH), 2920vs, 2852s ν(CH), 1623s ν(CC + CN), 1244vs $\nu_{as}(SO_3)$, 1035m $\nu_s(SO_3)$, 831m γ(CH). δ_H (300 MHz; $CDCl_3$; $SiMe_4$): 0.88 (6 H, t, $^3J_{H-H}$ 6.8, CH_3), 1.30 (20 H, m, CH_2), 1.79 (4 H, m, CH_2), 3.99 (4 H, t, $^3J_{H-H}$ 6.4, OCH_2), 5.62 (CH_2Cl_2), 6.80 (1 H, s, CH), 7.00 (4 H, d, $^3J_{H-H}$ 8.8, H_m), 7.69 (4 H, d, $^3J_{H-H}$ 8.7, H_o).

OTf-4,12 (22): light brown solid (45%). Found: C, 61.4; H, 7.1; N, 4.5; S, 5.0%. $C_{31}H_{45}N_2O_2CF_3SO_3$ requires C, 61.3; H, 7.2; N, 4.5; S, 5.1%. ν_{max}/cm^{-1}: 3163m ν(NH), 2919s, 2851s ν(CH), 1623s ν(CC + CN), 1240vs $\nu_{as}(SO_3)$, 1034s $\nu_s(SO_3)$, 825s γ(CH). δ_H (300 MHz; $CDCl_3$; $SiMe_4$): 0.88 (6 H, t, $^3J_{H-H}$ 6.7, CH_3), 1.27 (20 H, m, CH_2), 1.81 (4 H, m, CH_2), 4.02 (4 H, t, $^3J_{H-H}$ 6.3, OCH_2), 6.86 (1 H, s, CH), 7.03 (4 H, d, $^3J_{H-H}$ 8.8, H_m), 7.67 (4 H, d, $^3J_{H-H}$ 8.8, H_o).

OTf-8,12 (23): light brown solid (46%). Found: C, 63.7; H, 7.6; N, 4.2; S, 4.4%. $C_{35}H_{53}N_2O_2CF_3SO_3{\cdot}0.2CH_2Cl_2$ requires C, 63.3; H, 7.8; N, 4.1; S, 4.7%. ν_{max}/cm^{-1}: 3154m ν(NH), 2918s, 2849m ν(CH), 1624s ν(CC + CN), 1240vs $\nu_{as}(SO_3)$, 1035s $\nu_s(SO_3)$, 826vs γ(CH). δ_H (300 MHz; $CDCl_3$; $SiMe_4$): 0.88 (6 H, t, $^3J_{H-H}$ 6.7, CH_3), 1.27 (28 H, m, CH_2), 1.81 (4 H, m, CH_2), 4.02 (4

H, t, $^3J_{H-H}$ 6.3, OCH_2), 5.62 (CH_2Cl_2), 6.86 (1 H, s, CH), 7.03 (4 H, d, $^3J_{H-H}$ 8.8, H_m), 7.67 (4 H, d, $^3J_{H-H}$ 8.8, H_o), 14.21 (2 H, b, NH). δ_F (282.40 MHz; $CDCl_3$): –78.58

[$H_2pz^{R(4)R(12)}$]: white solid (49%). Found: C, 77.7; H, 3.0(> LS); N, 5.8%. $C_{31}H_{44}N_2O_2$ requires C, 78.1; H, 3.3; N, 5.9%. ν_{max}/cm^{-1}: 3229m ν(NH), 2923vs, 2869m, 2850s ν(CH), 1617m ν(CN), 1507s ν(CC), 790s γ(CH). δ_H (300 MHz; $CDCl_3$; $SiMe_4$): 0.88 (3 H, t, $^3J_{H-H}$ 6.0, CH_3'), 0.99 (3 H, t, $^3J_{H-H}$ 6.8, CH_3), 1.26 (20 H, m, CH_2), 1.80 (4 H, m, CH_2), 4.01 (4 H, t, $^3J_{H-H}$ 6.4, OCH_2), 6.70 (1 H, s, CH), 6.95 (4 H, m, H_m + $H_{m'}$), 7.65 (2 H, d, $^3J_{H-H}$ 8.5, H_o), 8.07 (2 H, d, $^3J_{H-H}$ 8.4, $H_{o'}$).

[$H_2pz^{R(8)R(12)}$]: white solid (52%). Found: C, 78.5; H, 9.4(> LS); N, 5.1%. $C_{35}H_{52}N_2O_2$ requires C, 78.9; H, 9.8; N, 5.3%. ν_{max}/cm^{-1}: 3429m ν(NH), 2920vs, 2852s ν(CH), 1615m ν(CN), 1504s ν(CC), 791s γ(CH). δ_H (300 MHz; $CDCl_3$; $SiMe_4$): 0.88 (6 H, t, $^3J_{H-H}$ 6.3, CH_3), 1.26 (28 H, m, CH_2), 1.79 (4 H, m, CH_2), 3.97 (4 H, t, $^3J_{H-H}$ 6.5, OCH_2), 6.68 (1 H, s, CH), 6.91 (4 H, d, $^3J_{H-H}$ 8.6, H_m), 7.63 (4 H, d, $^3J_{H-H}$ 8.6, H_o).

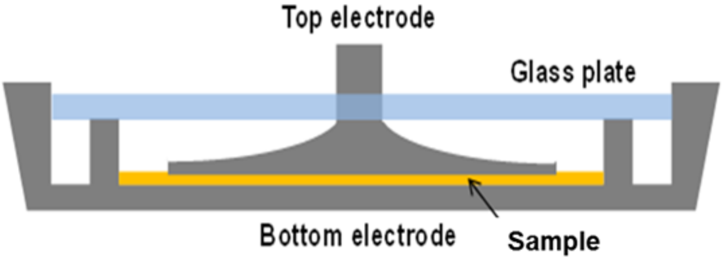

Figure S1. Custom-built liquid-solid measurement cell to obtain the conductivity and dielectric properties by impedance spectroscopy in the powder and liquid-crystalline state between the top and bottom electrodes.

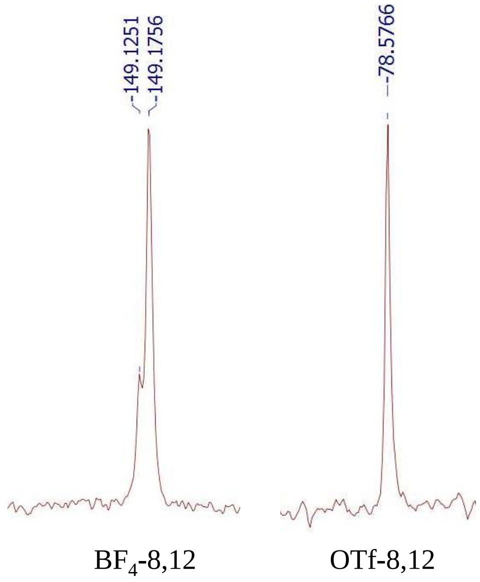

Figure S2. ^{19}F-NMR spectra of compounds BF$_4$-8,12 (10) and OTf-8,12 (23) in CDCl$_3$ solution at room temperature.

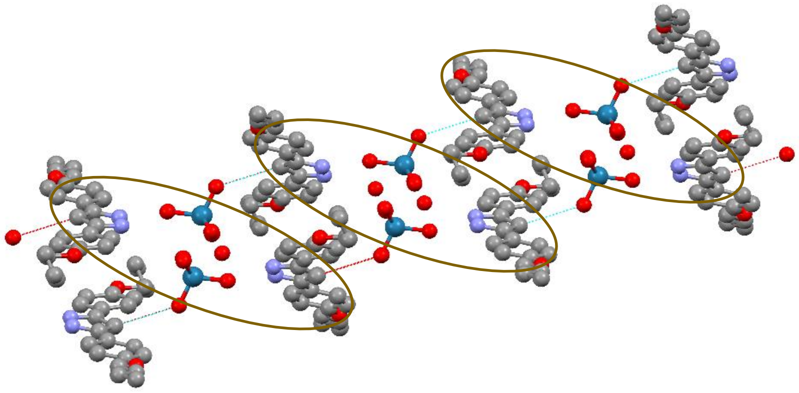

Figure S3. Packing of ReO$_4$-4,4·H$_2$O (11·H$_2$O): view of the layer in the *ac* plane (the double columns are marked by brown circles)

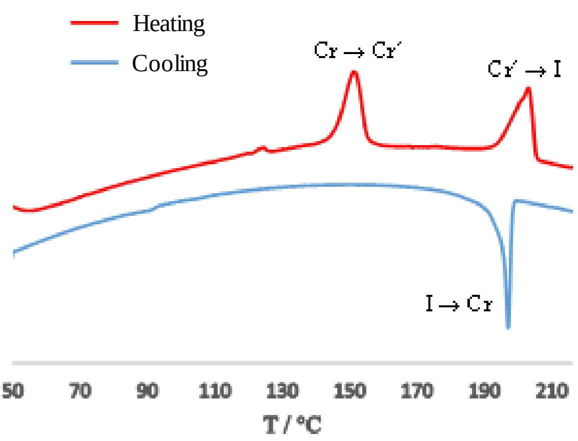

Figure S4. DSC thermogram of the OTf-4,12 specie (22)

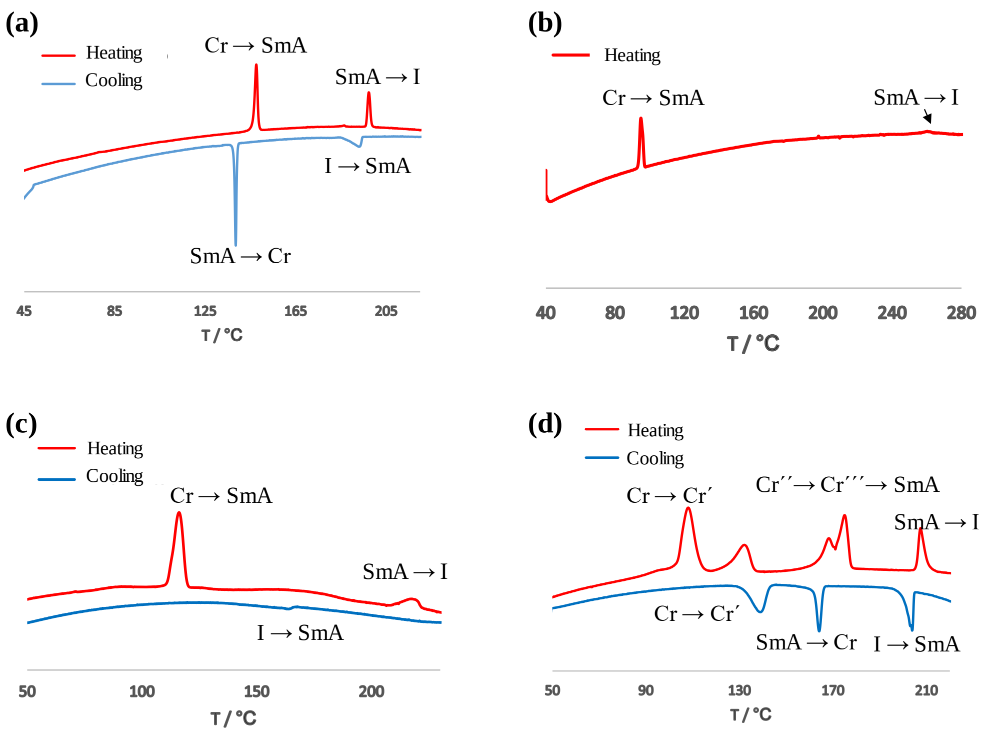

Figure S5. DSC thermograms of species Cl-8,8 (2) (a), ReO_4-8,8 (12) (b), BF_4-8,8 (7) (c) and PTS-4,12 (18) (d)

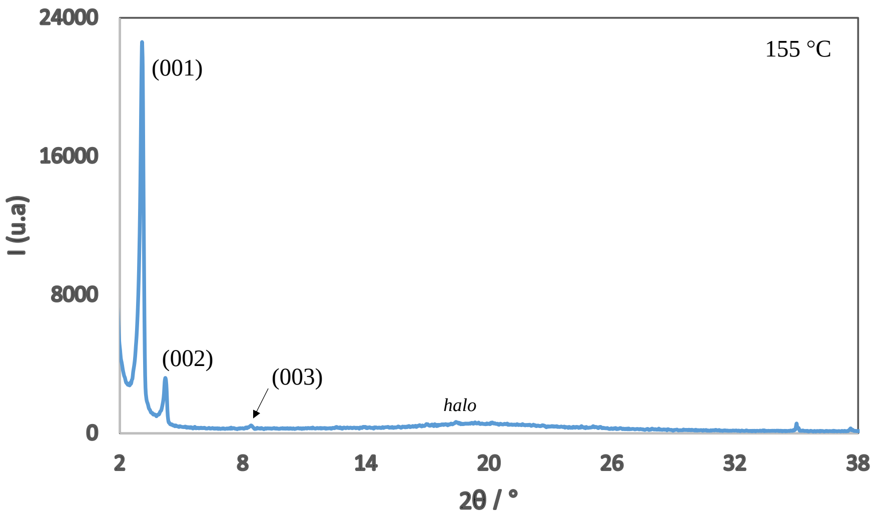

Figure S6. Diffractogram of compound PTS-8,8 (16) at 155 °C on heating.

Table S1. Crystal and refinement data for ReO_4-4,4·H_2O (11·H_2O)

Empirical formula	[$C_{23}H_{31}N_2O_7Re$]
Formula weight	660.76
Crystal system	Monoclinic
Space group	*C*2/*c*
Space group number	15
a / Å	18.928(2)
b / Å	14.4595(16)
c / Å	20.365(2)
β (°)	115.513(2)
V / Å^3	5030.2(10)
Z	8
T / K	293(2)
F(000)	2512
ρ_c / g cm^{-3}	1.674
μ / mm^{-1}	4.875
Scan technique	ω and φ
Data collected	(-22, -17, -24) to (22, 15, 22)
θ range (°)	1.85 to 25.50
Reflections collected	19699
Independent reflections	4668 (R_{int} = 0.0906)
Completeness to maximum θ (%)	99.5
Data / restraints / parameters	4668 / 8 / 294
Observed reflections [$I > 2\sigma(I)$]	2345
R[1]	0.0533
R_{wF}[2]	0.1796

[1] $\Sigma[|F_o| - |F_c|] / \Sigma[|F_o|]$. [2] $\{\Sigma[w(F_o^2 - F_c^2)^2] / \Sigma[w(F_o^2)^2]\}^{1/2}$

Table S2. Distances (Å) and bond angles (°) selected from the compound (ReO_4-4,4·H_2O) (11·H_2O)

Bond	Distance (Å)	Bonds	Angle (°)
N1–N2	1.35(2)	N1–N2–C3	109(2)
N1–C5	1.34(2)	N2–N1–C5	109(2)
N2–C3	1.34(2)	N2–C3–C4	108(2)
C3–C4	1.35(2)	N1–C5–C4	106(2)
C4–C5	1.42(2)	C3–C4–C5	107(2)
C3–C6	1.46(2)	N2–C3–C6	121(2)
C5–C12	1.46(2)	N1–C5–C12	124(2)
Re–O (mean)	1.65	O–Re–O (mean)	109.7

Table S3. Phase transitions for the salts [$H_2pz^{R(n)R(m)}$] [OTf] (20 – 23) determined by DSC

Compound	Transition[a]	T^b / °C	ΔH / kJ mol^{-1}
OTf-4,4 (20)	Cr → Cr′	160	3.3
	Cr′ → I	228	22
OTf-8,8 (21)	Cr → Cr′	128[c]	2.8
	Cr′ → Cr′′	147[c]	3.1
	Cr′′→ Cr′′′	160[c]	13.5
	Cr′′′→ I	246[c]	8.3
OTf-4,12 (22)	Cr → Cr′	145	19.2
	Cr′ → I	195	17.7
OTf-8,12 (23)	Cr → Cr′	133[c]	13.4
	Cr′ → Cr′′	161[c]	2.2
	Cr′′ → I	215	8.8

[a] Cr, Cr′, Cr′′, Cr′′′: solid phases, I: isotropic liquid; [b] Onset temperature determined by DSC; [c] Temperature of the DSC peak
